# Protective **α**1-antitrypsin effects in autoimmune vasculitis are compromised by methionine oxidation

**DOI:** 10.1172/JCI160089

**Published:** 2022-12-01

**Authors:** Maximilian Ebert, Uwe Jerke, Claudia Eulenberg-Gustavus, Lovis Kling, Dieter Jenne, Marieluise Kirchner, Philipp Mertins, Markus Bieringer, Saban Elitok, Kai-Uwe Eckardt, Adrian Schreiber, Alan D. Salama, Ralph Kettritz

**Affiliations:** 1 Experimental and Clinical Research Center, a cooperation between the Max Delbrück Center for Molecular Medicine in the Helmholtz Association and Charité — Universitätsmedizin Berlin, Berlin, Germany.; 2 Charité — Universitätsmedizin Berlin, corporate member of Freie Universität Berlin and Humboldt-Universität zu Berlin, Berlin, Germany.; 3 Max Delbrück Center for Molecular Medicine in the Helmholtz Association, Berlin, Germany,; 4 Department of Nephrology and Medical Intensive Care, Charité — Universitätsmedizin Berlin, Germany.; 5 Max-Planck-Institute of Neurobiology, Planegg-Martinsried, Germany.; 6 Core Unit Proteomics, Berlin Institute of Health at Charité — Universitätsmedizin Berlin and Max Delbrück Center for Molecular Medicine, Berlin, Germany.; 7 Department of Nephrology, Helios Klinikum Berlin-Buch, Berlin, Germany.; 8 Department of Nephrology and Endocrinology, Ernst von Bergmann Klinikum, Potsdam, Germany.; 9 University College London, Royal Free Hospital, London, United Kingdom.

**Keywords:** Autoimmunity, Inflammation, Innate immunity, Vasculitis

## Abstract

**Background:**

Antineutrophil cytoplasmic autoantibody–associated (ANCA-associated) vasculitidies (AAV) are life-threatening systemic autoimmune conditions. ANCAs directed against proteinase 3 (PR3) or myeloperoxidase (MPO) bind their cell surface-presented antigen, activate neutrophils, and cause vasculitis. An imbalance between PR3 and its major inhibitor α1-antitrypsin (AAT) was proposed to underlie PR3- but not MPO-AAV. We measured AAT and PR3 in healthy individuals and patients with AAV and studied protective AAT effects pertaining to PR3- and MPO-ANCA.

**Methods:**

Plasma and blood neutrophils were assessed for PR3 and AAT. WT, mutant, and oxidation-resistant AAT species were produced to characterize AAT-PR3 interactions by flow cytometry, immunoblotting, fluorescence resonance energy transfer assays, and surface plasmon resonance measurements. Neutrophil activation was measured using the ferricytochrome C assay and AAT methionine-oxidation by Parallel Reaction Monitoring.

**Results:**

We found significantly increased PR3 and AAT pools in patients with both PR3- and MPO-AAV; however, only in PR3-AAV did the PR3 pool correlate with the ANCA titer, inflammatory response, and disease severity. Mechanistically, AAT prevented PR3 from binding to CD177, thereby reducing neutrophil surface antigen for ligation by PR3-ANCA. Active patients with PR3-AAV showed critical methionine-oxidation in plasma AAT that was recapitulated by ANCA-activated neutrophils. The protective PR3-related AAT effects were compromised by methionine-oxidation in the AAT reactive center loop but preserved when 2 critical methionines were substituted with valine and leucine.

**Conclusion:**

Pathogenic differences between PR3- and MPO-AAV are related to AAT regulation of membrane-PR3, attenuating neutrophil activation by PR3-ANCA rather than MPO-ANCA. Oxidation-resistant AAT could serve as adjunctive therapy in PR3-AAV.

**FUNDING:**

This work was supported by KE 576/10-1 from the Deutsche Forschungsgemeinschaft, SCHR 771/8-1 from the Deutsche Forschungsgemeinschaft, grant 394046635 — SFB 1365 from the Deutsche Forschungsgemeinschaft, and ECRC grants.

## Graphical abstract

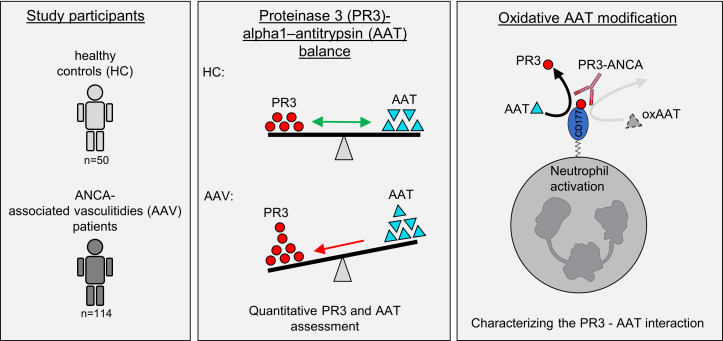

## Introduction

Antineutrophil cytoplasmic autoantibody–associated (ANCA-associated) vasculitis (AAV) comprises a group of life-threatening, systemic autoimmune diseases, most frequently affecting the lungs and kidneys (1). The majority of patients with the clinical syndrome of granulomatosis with polyangiitis (GPA) have ANCA to proteinase 3 (PR3), whereas most patients with microscopic polyangiitis (MPA) have ANCA directed against myeloperoxidase (MPO). The ANCA autoantigens are exclusively expressed by myeloid cells, with the highest amounts in neutrophils. Mechanistically, ANCA binding to their cell surface–presented target autoantigens initiates neutrophil activation and subsequent vascular inflammation and injury (2). PR3 has a typical bimodal cell membrane pattern (herein referred to as mPR3) with distinct mPR3^lo^ and mPR3^hi^ neutrophil subsets (3). The percentage of the mPR3^hi^ subset varies between 0% and 100% in the population, is genetically determined, and is stable in each individual (4–6). mPR3^hi^ neutrophils respond more vigorously to PR3-ANCA–induced activation (7), and a large mPR3^hi^ neutrophil subset represents a risk factor for AAV and worse clinical outcomes (4, 6, 8). The bimodal mPR3 pattern is a consequence of the epigenetically controlled, subset-restricted expression of CD177 (9).The bimodal mPR3 pattern is a consequence of the epigenetically controlled, subset-restricted expression of CD177 (9). CD177 functions as a high-affinity PR3 receptor yielding distinct CD177^–^/mPR3^lo^ and CD177^+^/mPR3^hi^ neutrophils (10, 11). While the neutrophil-activating effects of both PR3- and MPO-ANCA have been established in vitro, the pathogenicity of only MPO-ANCA has been confirmed in animal models, because of the lack of convincing PR3-ANCA models (12–18).

The clinical syndromes — GPA and MPA — and the ANCA specificities — to PR3 and MPO — result in distinct clinical characteristics, while the differences in underlying pathogenic mechanisms remain less clearly defined (19, 20). PR3, the autoantigen in PR3-AAV, is a member of the neutrophil serine protease (NSP) family with pathogenic implications in both AAV entities (21–24). α1-antitrypsin (AAT) is the major PR3 inhibitor, and recent genome-wide association studies (GWAS) found strong GPA- and PR3-AAV associations with SNPs near or within the genes for PR3 (*PRTN3)* and AAT (*SERPINA1*) (25, 26). These genetic associations were not seen in patients with MPO-AAV, suggesting that PR3- and MPO-AAV are genetically distinct diseases. *PRTN3* SNPs from the AAV GWAS provided an expression quantitative trait loci (eQTL) affecting neutrophil PR3 transcription (26) and a protein quantiative trait loci (pQTL) that associated with increased plasma PR3 levels in healthy controls (HCs) (27). The GWAS-identified *SERPINA1* SNP in PR3-AAV is in linkage disequilibrium with the *SERPINA1* null (z) allele that leads to lower plasma AAT (26–28). Based on these findings, mechanistic concepts regarding disease pathogenesis were proposed suggesting roles for increased PR3 and/or reduced AAT in PR3-AAV, but not in MPO-AAV (25–27).

Because systematic studies investigating variations in levels of PR3 and AAT in AAV are lacking, we prospectively evaluated PR3 and AAT in HC and patients with AAV and explored clinical correlations. Our patient-related data led us to experimentally characterize protective AAT effects targeting mPR3 on neutrophils, thereby reducing neutrophil activation by PR3-ANCA. We also identified critical disease modifiers of the PR3-AAT interaction that diminished the protective AAT effects and tested an AAT variant that resisted this loss-of-function modification.

## Results

### Plasma PR3, AAT, and the PR3:AAT ratio are significantly increased in patients with active PR3- and MPO-AAV.

**Figure 1:**
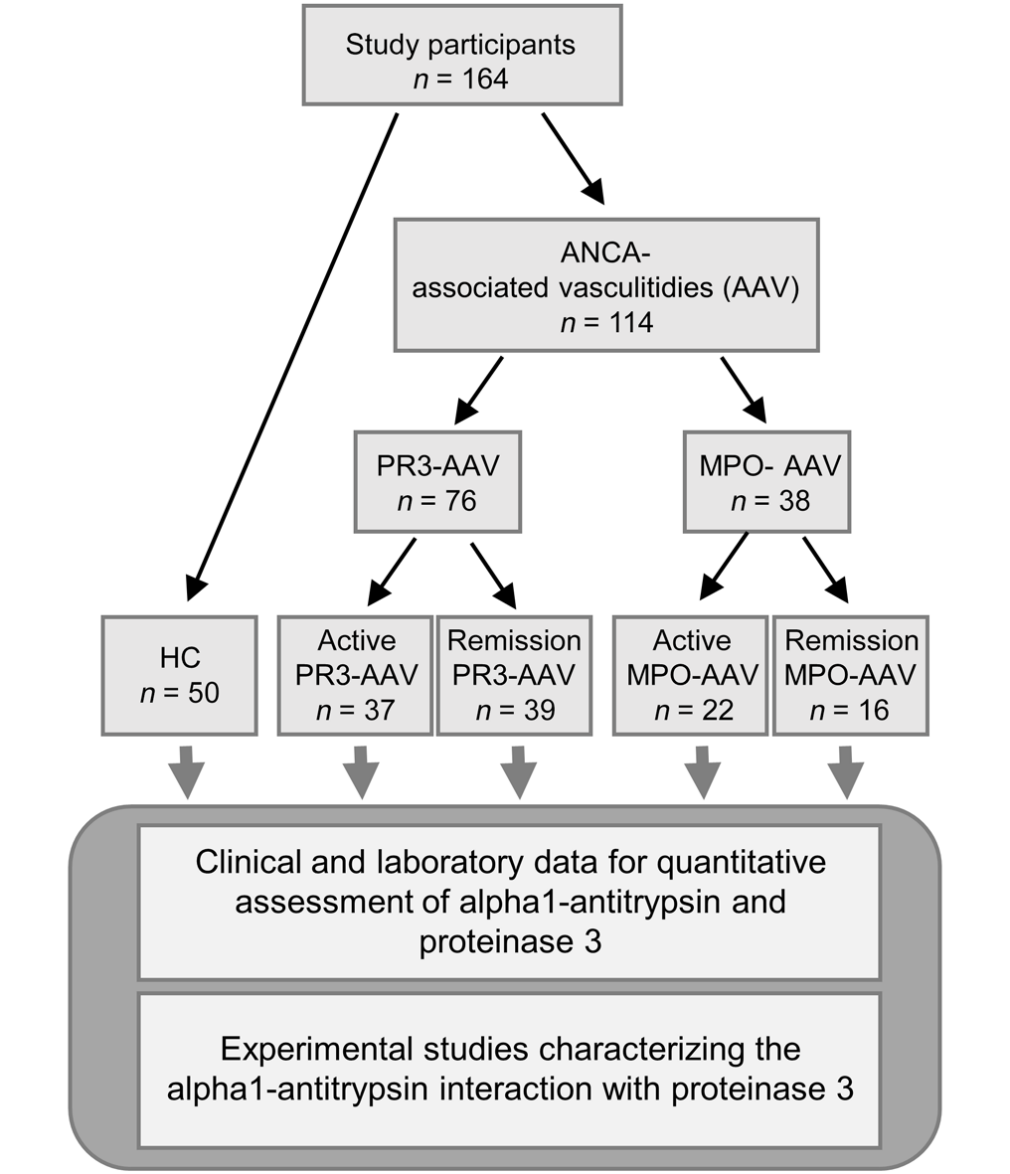
Patients and study design. HCs and patients with AAV were recruited for assessment of PR3 and AAT.

**Figure 2:**
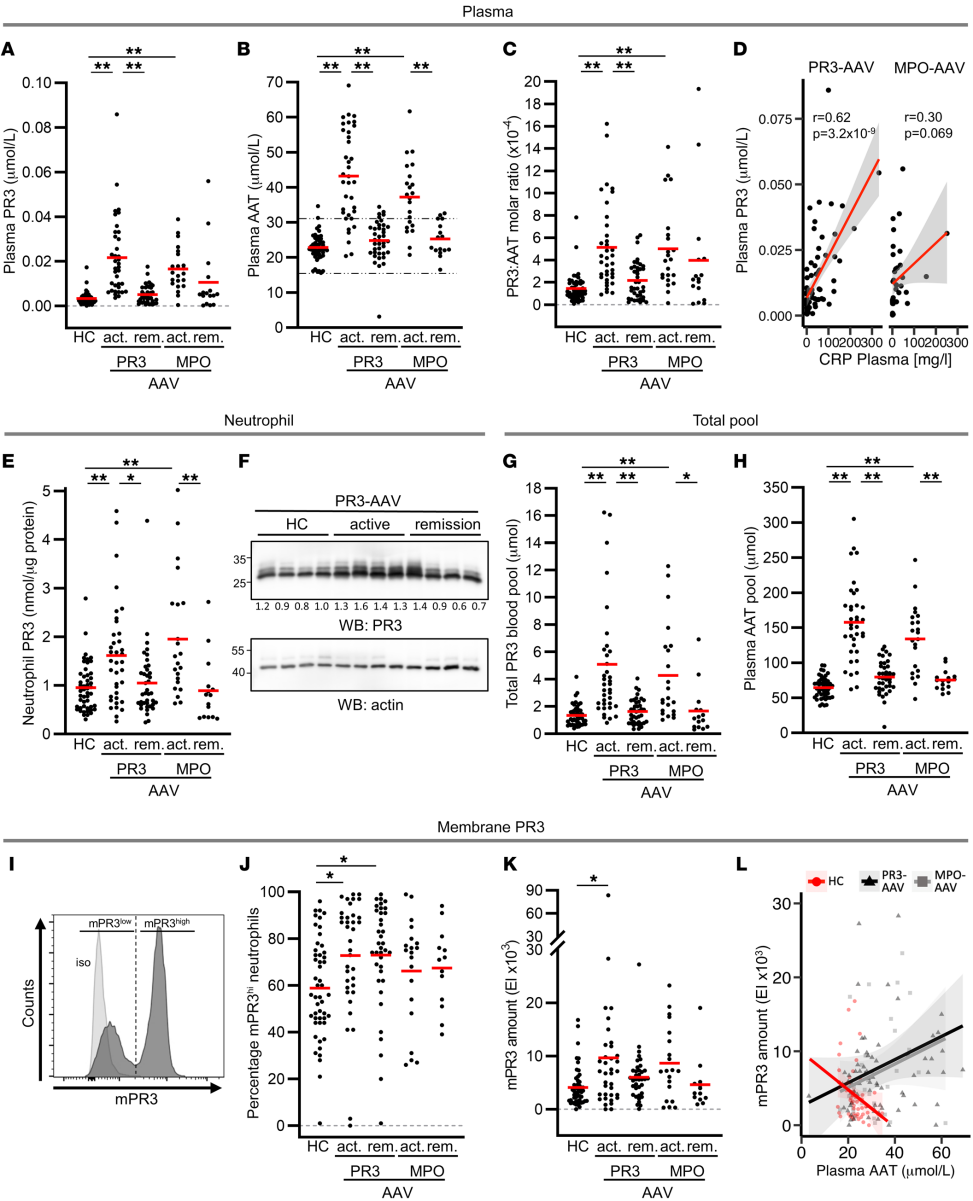
PR3 and AAT are both increased in patients with active PR3- and MPO-AAV and normalize with remission. Plasma from HCs, and patients with active (act) and remission (rem) PR3- and MPO-AAV were analyzed for (**A**) plasma PR3 (*n* = 50, 37, 39, 22, 16 from left to right) and (**B**) plasma AAT (*n* = 50, 36, 39, 22, 16 from left to right). The normal AAT concentration between 16.2 and 30.2 μmol/L(28) is indicated by the dotted black lines. Red lines indicate the mean. (**C**) The plasma PR3 to AAT molar ratio was calculated (*n* = 50, 36, 39, 22, 16 from left to right). (**D**) Plasma PR3 correlation with plasma CRP in PR3- and MPO-AAV is illustrated by the red regression lines and the gray shadings indicating the 95% confident intervals. (**E**) Neutrophil PR3 was assessed in HC and patients with active PR3- and MPO-AAV by ELISA (*n* = 50, 37, 39, 22, 15 from left to right), and (**F**) by immunoblotting in randomly selected HCs and patients with PR3-AAV. Densitometry measurements of single PR3 bands normalized to a β-actin loading control are indicated. (**G**) The total PR3 blood pool (*n* = 50, 37, 39, 22, 15 from left to right) and (**H**) plasma AAT pool (*n* = 50, 36, 39, 22, 16 from left to right) was calculated in HCs and patients with AAV. Neutrophils from HCs and patients with AAV were stained with an anti-PR3 mAb and analyzed by flow cytometry. (**I**) A typical histogram illustrates the bimodal staining pattern with distinct mPR3^lo^ and PR3^hi^ neutrophil subsets. (**J**) The percentage of mPR3^hi^ neutrophils is depicted (*n* = 50, 36, 37, 20, 13 from left to right). (**K**) The mPR3 amount is given as expression index of the MFI (MFI EI, *n* = 50, 36, 37, 20, 13 from left to right). (**L**) Spearman correlation between mPR3 amount and plasma AAT is illustrated. HC are depicted as red circles (r = –0.32, *P =* 0.02), all patients with PR3-AAV as black triangles (r = 0.24, *P =* 0.04), and all patients with MPO-AAV as gray squares (r=0.23, *P =* 0.21). Individual results are depicted, and the regression line for each color-coded group is given by the solid lines together with the shaded areas indicating the 95% confident intervals. 1-way ANOVA was performed with Tukey’s posthoc testing. *P < 0.05, ** *P* < 0.01.

We recruited 50 HC and 114 patients with AAV (37 patients with active PR3-ANCA and 39 patients with PR3-ANCA in remission, and 22 patients with active MPO-ANCA and 16 patients with MPO-ANCA in remission) from 3 hospitals between January 2020 and July 2021 for assessment of PR3 and AAT. A flow diagram outlining the study design and the participating patients is depicted in Figure 1. HC and patient characteristics are listed in Supplemental Table 1 (supplemental material available online with this article; https://doi.org/10.1172/JCI160089DS1). Plasma PR3 and AAT levels were significantly increased in both patients with active PR3- and MPO-AAV compared with HC, and both values normalized with disease remission (Figure 2, A and B). Only 1 patient with AAV in remission showed strongly decreased AAT values, suggesting the presence of a homozygous *SERPINA1* z allele. Increased PR3 protein levels exceeded increased AAT, leading, on average, to a 3.3-fold higher plasma PR3:AAT molar ratio in active disease (Figure 2C). Plasma PR3 levels correlated with c-reactive protein in PR3-AAV but not in MPO-AAV (Figure 2D). Plasma AAT levels correlated with the inflammation marker CRP in PR3- and MPO-AAV (Supplemental Figure 1A).

### Plasma, neutrophil, and total PR3 blood pools are increased in patients with active AAV and correlate with inflammation, vascular injury, and PR3 autoimmunity in PR3-AAV but not MPO-AAV.

We measured PR3 protein in lysates from blood neutrophils by ELISA and found that neutrophils from patients with active PR3-AAV contained 1.7-fold more PR3 than lysates from HCs, and patients with active MPO-AAV had 2.0-fold more PR3 compared to HCs (Figure 2E). PR3 immunoblots showed similar PR3 band patterns in patients with PR3-AAV and HCs, indicating that AAV-associated glycosylation did not differ between patients and HCs (Figure 2F). PR3 transcription is silenced in mature blood neutrophils but is reactivated in active AAV (29–31). We observed that PR3, but not AAT transcription, was significantly increased in neutrophils from patients with active PR3-AAV compared with HCs and patients in remission (Supplemental Figure 1, B and C).

We calculated the PR3 antigen pool in plasma (PR3 concentration × estimated plasma volume), in neutrophils (PR3 neutrophil content × blood neutrophil count), and, taken together, the total PR3 blood pool. The neutrophil PR3 pool was approximately 100 × larger compared with the plasma PR3 pool, but both pools increased significantly in patients with active AAV and normalized with patients in remission (Supplemental Figure 1D and E). Hence, the total PR3 blood pool was strongly increased in patients with active AAV (3.8-fold in PR3-AAV and 3.2-fold in MPO-AAV) (Figure 2G). We observed significant positive correlations of the total PR3 blood pool with clinical markers of systemic inflammation, such as CRP; anemia, such as hemoglobin; and kidney injury, such as creatinine and erythrocyturia, in patients with PR3-AAV but not in patients with MPO-AAV (Table 1). Similar correlations were seen for the plasma and the neutrophil PR3 pool individually. The plasma PR3 pool, but not the total PR3 blood and neutrophil pool correlated with autoimmunity (PR3-ANCA titer). The AAT plasma pool was also increased in patients with both ANCA specificities (Figure 2H).

### PR3 is increased on the cell membrane of neutrophils from patients with PR3-AAV.

Because PR3-ANCA binding to mPR3 initiates neutrophil activation, we assessed mPR3 on isolated neutrophils from HCs and patients with AAV. A representative flow cytometry histogram illustrates the typical bimodal mPR3 staining (Figure 2I). We analyzed our cohort with respect to both the portion of mPR3^hi^ neutrophils and to the amount of mPR3 — as assessed by the mean fluorescence intensity (MFI). Compared with HCs, the portion of mPR3^hi^ neutrophils was increased in patients with both active and remission PR3-AAV, with a similar trend for MPO-AAV — supporting the notion that this AAV risk parameter is genetically determined (Figure 2J) (5, 6, 32). In contrast, mPR3 amounts were significantly increased in patients with active PR3-AAV, but not those in remission, indicating that inflammatory diseases elevate the amount of PR3 on the neutrophil surface without changing the percentage of mPR3^hi^ neutrophils (Figure 2K). Parallel assessment of mCD177 in the HC and patient groups showed the same pattern (Supplemental Figure 1, F and G). In HCs, the mPR3 amount showed an inverse correlation with plasma AAT that was not observed in patients with AAV (Figure 2L). These observations led the authors to hypothesize that extracellular AAT reduced mPR3, but that this effect was compromised in AAV, despite increased AAT levels.

### Recombinant WT, but not mutant AAT binds and neutralizes proteolytically active PR3, and releases PR3 from the neutrophil membrane in a reversible manner.

**Figure 3:**
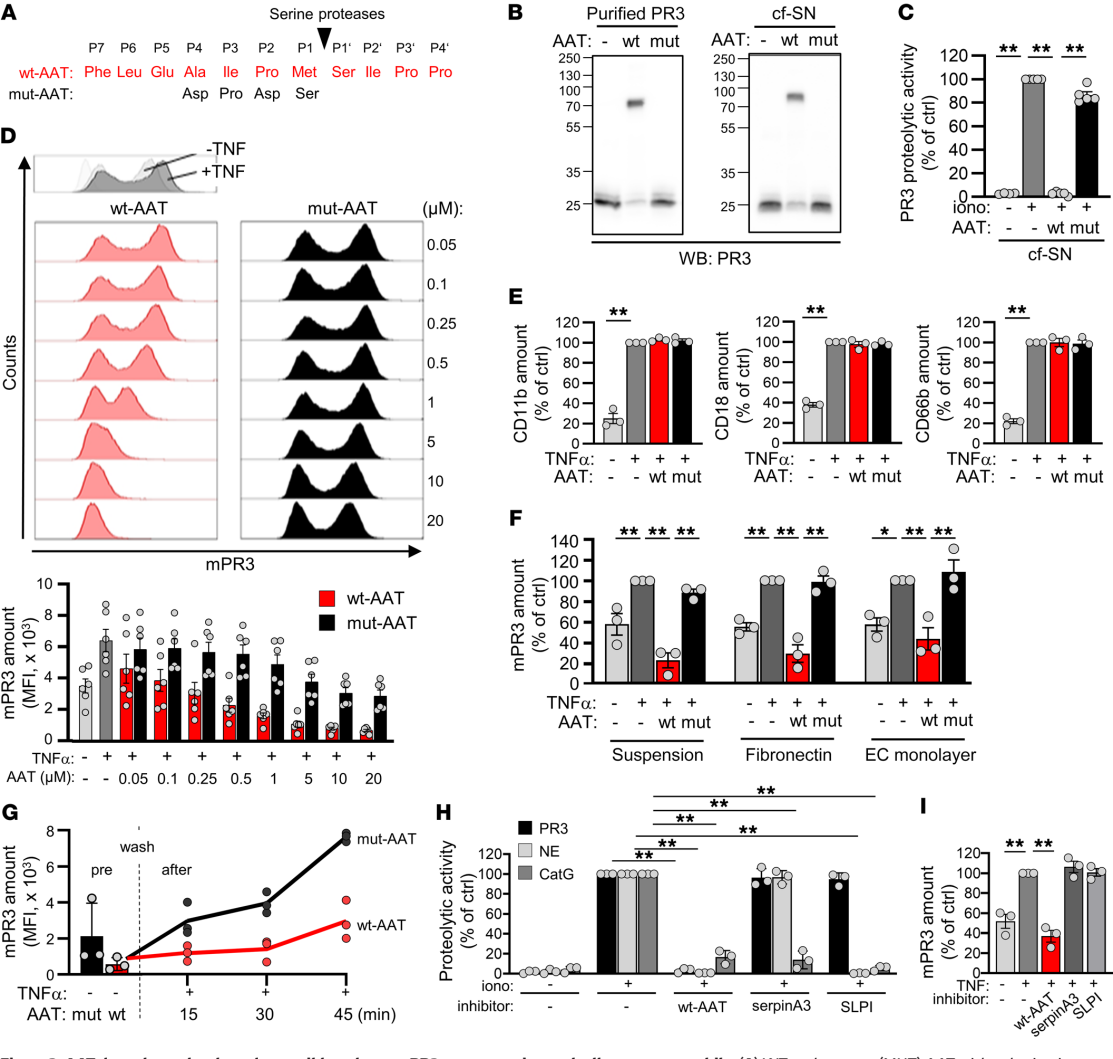
AAT dose-dependently and reversibly reduces mPR3 on suspension and adherent neutrophils. (**A**) WT and mutant (MUT) AAT with substitutions in the reactive center loop were produced. (**B**) WT- or MUT-AAT were incubated with purified PR3 or cfSN from ionophore A23187-activated neutrophils and assessed by immunoblotting using an anti-PR3 mab. WT-AAT but not MUT-AAT formed the typical 72 kDa AAT:PR3 complex. A typical of 3 experimental replicates is shown. (**C**) Incubation of cfSN from ionophore A23187-activated neutrophils with 0.25 μM WT-AAT but not MUT-AAT neutralized the proteolytical PR3 activity using a PR3-specific FRET substrate (*n* = 5/group). (**D**) TNF-α priming increased mPR3 on isolated neutrophils as shown in a typical flow cytometry histogram. Incubation of 1 × 10^6^ primed neutrophils with the indicated WT-AAT but not MUT-AAT concentrations for 30 minutes decreased mPR3 staining in a dose-dependent manner. A typical experiment together with the corresponding statistics is depicted (*n* = 6/group). (**E**) Incubation of primed neutrophils with 0.25 μM WT-AAT did not decrease neutrophil surface staining for CD11b, CD18, and CD66b by flow cytometry (*n* = 3/group). (**F**) Adding 0.25 μM WT-AAT but not MUT-AAT decreased mPR3 on primed neutrophils in suspension, adherent to fibronectin, and adherent to a gMVEC monolayer (EC monolayer), respectively (*n* = 3/group). (**G**) Isolated neutrophils were incubated with 0.25 μM WT- or MUT-AAT for 30 minutes (pre), followed by washing to remove AAT (wash). In the presence of newly added 0.25 μM MUT- but not WT-AAT, mPR3 reoccurred on the surface of primed neutrophils over a 45 minute time period (*n* = 3/group). Effect of WT-AAT, serpinA3, and SLPI on (**H**) proteolytic activity of PR3, NE, and CatG using specific FRET substrates, and on (**I**) neutrophil mPR3 by flow cytometry. Individual results and the means ± SEM are given. For statistical analysis, 1-way ANOVA was performed with Tukey’s post-hoc testing. *P < 0.05, **P < 0.01.

To test our hypothesis, we produced recombinant WT–AAT and, as an important control protein, a mutant form (MUT-AAT) that we had recently generated (33) containing amino acid substitutions in the reactive center loop (Figure 3A). As expected, WT-AAT but not MUT-AAT bound purified PR3 and PR3 in cell-free supernatants (cfSNs) from activated neutrophils, as indicated by the formation of the typical 72-kDa PR3:AAT complex in anti-PR3 immunoblots (Figure 3B). Consequently, WT-AAT but not MUT-AAT abrogated the PR3-specific proteolytic fluorescence resonance energy transfer (FRET) activity in cfSNs (Figure 3C). Importantly, neutrophil incubation with increasing concentrations of WT-AAT dose-dependently reduced mPR3 on neutrophils, as determined by flow cytometry (Figure 3D). At higher concentrations, MUT-AAT somewhat reduced the MFI without affecting the typical bimodal mPR3 pattern, underscoring the importance of an appropriate control protein. WT-AAT did not reduce surface levels of CD11b, CD18, or CD66b (Figure 3E). Moreover, WT-AAT equally reduced mPR3 when neutrophils were incubated on fibronectin or on glomerular microvascular endothelial cells (gMVEC) (Figure 3F).

Neutrophils function not only in blood at high AAT concentrations, but also in inflamed tissues where AAT concentrations are significantly lower. We therefore assessed whether the mPR3 reduction by AAT was reversible under inflammatory conditions. Neutrophils were incubated with WT-AAT to reduce mPR3, washed to remove AAT, and then primed with TNFα. We observed a strong reappearance of mPR3 over time in the presence of MUT-AAT that was again suppressed by the WT-AAT (Figure 3G). Two other natural inhibitors of NSPs, namely serpinA3 and the chelonianin SLPI did not neutralize proteolytic PR3 activity or reduce mPR3, underscoring the importance of AAT in regulating mPR3 (Figure 3, H and I). These findings establish that AAT reduced mPR3 on the surface of neutrophils in a dose-dependent and reversible manner.

### AAT removes mPR3 from the neutrophil surface by preventing PR3 binding to CD177, thereby reducing PR3-ANCA–induced neutrophil activation.

**Figure 4:**
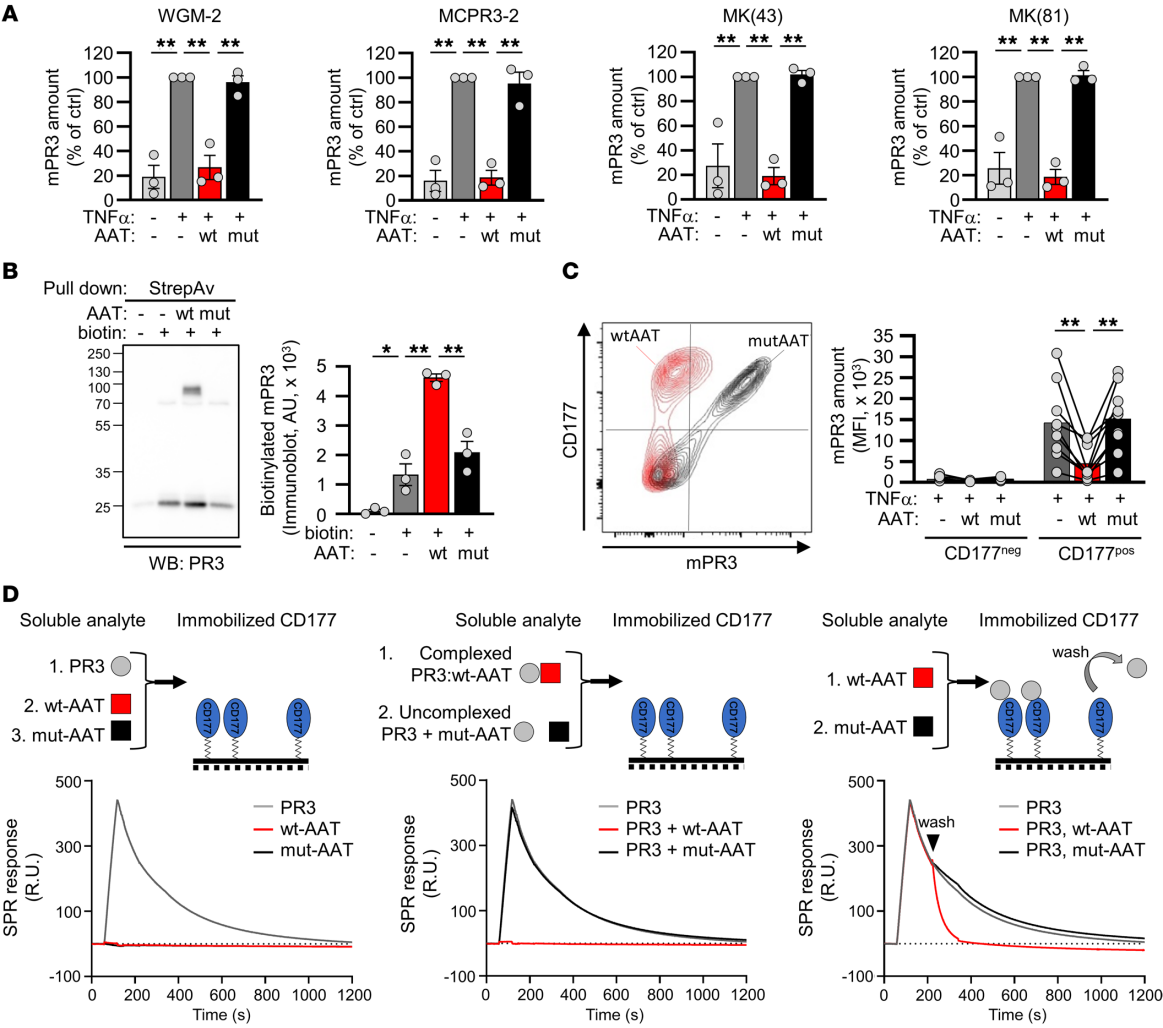
AAT reduces mPR3 on the neutrophil surface by competing with CD177 for PR3 binding, and subsequently ameliorates neutrophil activation by PR3-ANCA but not MPO-ANCA. (**A**) Isolated neutrophils were primed with TNFα, incubated with 0.25 μM WT- or MUT-AAT for 30 minutes, and mPR3 was stained using 4 different anti-PR3 mAbs (*n* = 3/group). By flow cytometry, all antibodies detected decreased mPR3 after neutrophil incubation with WT-AAT. Note that clone MCPR3-2 and WGM-2 recognize 2 different PR3 epitopes. (**B**) Surface proteins of isolated neutrophils were biotinylated prior to incubation with 0.25 μM WT- or MUT-AAT. Supernatants were collected after 30 minutes and biotinylated proteins were pulled down with streptavidin followed by electrophoresis and immunoblotting with an anti-PR3 mAb. A typical immunoblot and the corresponding statistics of 3 independent experiments are given. (**C**) Primed isolated neutrophils were incubated with 0.25 μM WT- or MUT-AAT for 30 minutes followed by double staining with mAbs to PR3 and CD177. A typical flow cytometry experiment together with the corresponding statistics, separately for the distinct CD177^–^ and CD177^+^ subsets, is given (*n* = 9/group). (**D**) The experimental setup together with the corresponding surface plasmon resonance sensorgrams are depicted. CD177 was immobilized to the sensor chip and the soluble analytes are indicated. High affinity–CD177 complex formation is observed with PR3, but not with WT- or MUT-AAT (left panel). Preincubating PR3 with WT-AAT but not MUT-AAT prevented PR3 from binding to immobilized CD177 (middle panel). Applying WT-AAT after the CD177:PR3 complex had formed displaced PR3 from CD177 (right panel). Individual results and the mean ± SEM are given. 1-way ANOVA (**A** and **B**) and repeated measures 1-way ANOVA (**C**) were performed with Tukey’s post-hoc testing. *P < 0.05, **P < 0.01.

Given the importance of mPR3 for PR3-ANCA binding and subsequent neutrophil activation, we explored the molecular mechanism by which AAT reduces mPR3. To exclude the possibility that AAT merely prevented antibody binding to PR3 by steric hindrance, mPR3 reduction by WT-AAT was confirmed using 3 additional mAbs to PR3, including clones MCPR3-2 and WGM-2 that recognize 2 different PR3 epitopes (Figure 4A) (34, 35). We then performed a mPR3 shedding assay after incubation of surface-biotinylated neutrophils with buffer, WT-, and MUT-AAT, respectively. We collected the cell-free supernatants (cfSNs) and performed a streptavidin-based pulldown of biotinylated proteins from these cfSNs followed by anti-PR3 immunoblotting. We found significantly more biotinylated mPR3 in the supernatants from WT-AAT-treated neutrophils, indicating that PR3 was indeed released from the neutrophil surface (Figure 4B).

The bimodal neutrophil mPR3 pattern is caused by subset-restricted expression of the PR3 receptor CD177, yielding distinct CD177^–^/mPR3^lo^ and CD177^+^/mPR3^hi^ neutrophils (9, 10). Flow cytometry after double-staining for PR3 and CD177 revealed that WT-AAT treatment reduced mPR3, particularly on the CD177^+^/mPR3^hi^ subset (Figure 4C). We hypothesized that AAT competes with CD177 for PR3 binding and performed surface plasmon resonance measurements (SPR) (Figure 4D). We observed that PR3, but not AAT, bound with a high affinity to immobilized CD177 (K_D_ = 20 × 10^–9^ M). PR3 preincubation with WT-AAT, but not with MUT-AAT, prevented PR3 binding to immobilized CD177. Importantly, CD177-bound PR3 dissociated from CD177 when WT-AAT was used as soluble analyte, but not MUT-AAT.

These data indicate that AAT reduced mPR3 on the surface of CD177^+^/mPR3^hi^ neutrophils by complexing with PR3, thereby preventing PR3 binding to CD177.

### Disease-associated modification of the AAT:PR3 interaction and its pathologic consequences.

**Figure 5:**
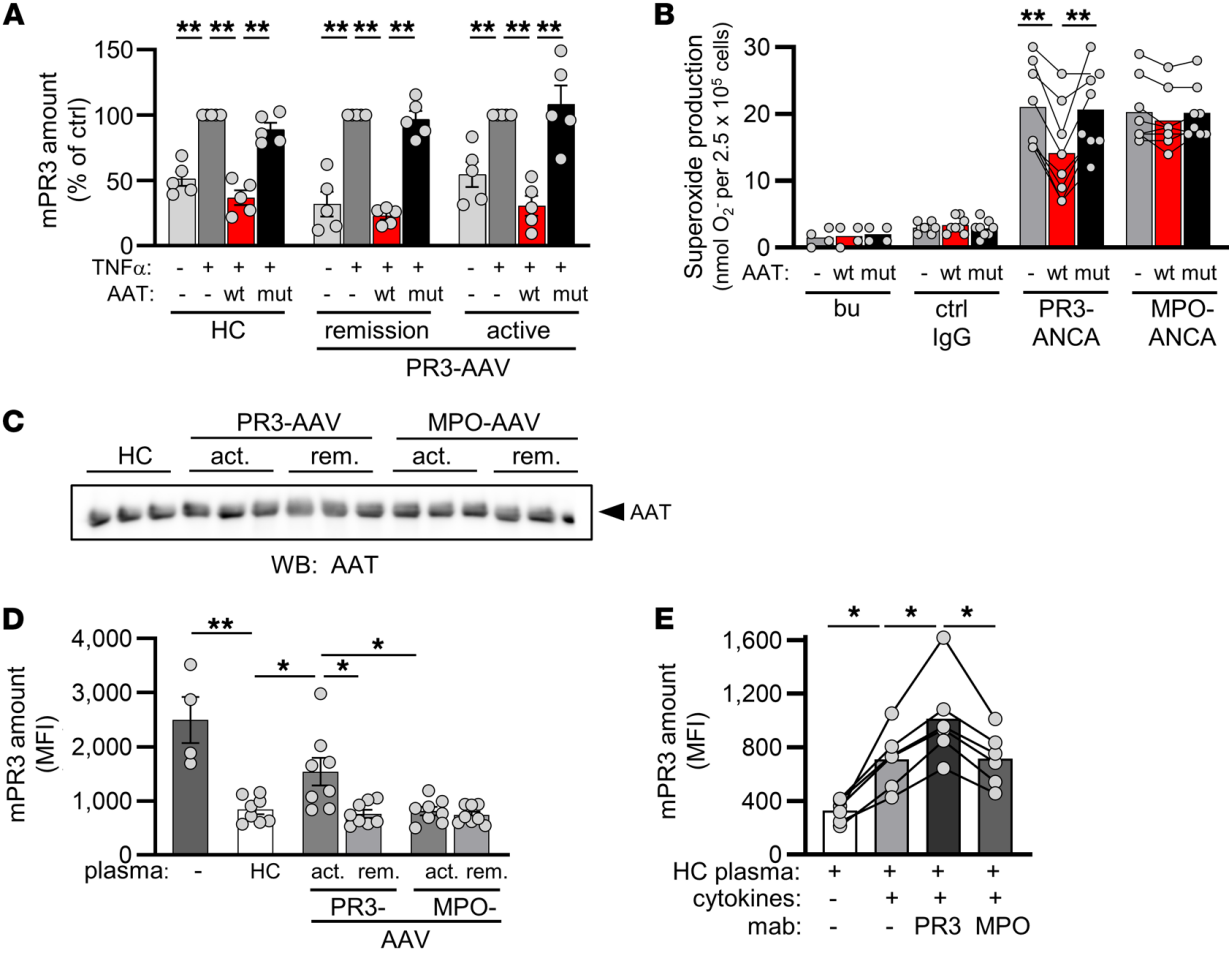
Disease-related modifiers of the mPR3-lowering AAT effect in the plasma of patients with PR3-AAV. (**A**) Neutrophils were isolated from HC, patients with active PR3-AAV, and patients with PR3-AAV in remission (*n* = 5/group). Primed neutrophils were incubated with buffer or 0.25 μM WT- or MUT-AAT for 30 minutes. By flow cytometry, WT-AAT significantly reduced mPR3 on neutrophils of all groups. (**B**) Primed neutrophils were incubated with 0.25 μM WT- or MUT-AAT for 30 minutes prior to the stimulation with human ANCA-IgG. Superoxide release was measured using the ferricytochrome C reduction assay (*n* = 4 independent experiments, each using 2 different ANCA preparations). (**C**) Plasma from HC and patients with AAV, selected for similar AAT concentrations, was diluted to achieve AAT of 0.25 μM and showed similar AAT band optical densities in AAT immunoblots. (**D**) HC neutrophils were incubated for 30 minutes in buffer or diluted plasma from HC or patient neutrophils as indicated. mPR3 was assessed by flow cytometry. The mPR3 reduction by plasma was significantly less with plasma from 8 different patients with active PR3-AAV (*n* = 4 independent experiments) (**E**) HC neutrophils were incubated in HC plasma as in **C**, or in HC plasma supplemented with a cytokine cocktail and mAbs to PR3 or MPO as indicated (*n* = 6/group). Individual results and the mean ± SEM are given. 1-way ANOVA (**A** and **D**) and repeated measures 1-way ANOVA within each group (**B** and **E**) were performed with Tukey’s posthoc testing. *P < 0.05, **P < 0.01.

We next explored disease-associated factors that modify mPR3, AAT, and possibly their interaction. We first compared the susceptibility of the neutrophils from HCs and patients with active AAV to mPR3 reduction by AAT. WT-AAT decreased mPR3 on neutrophils from HC, patients with active PR3-AAV, and patients with PR3-AAV in remission to a similar degree, excluding intrinsic neutrophil factors reducing the mPR3-lowering effect of AAT (Figure 5A). We then determined the consequence of AAT-mediated mPR3 reduction for ANCA-induced neutrophil activation by measuring NADPH oxidase-dependent neutrophil respiratory burst — a robust activation indicator. When neturophils from HCs were incubated with WT-AAT before stimulation with ANCA, superoxide production was significantly reduced in response to PR3-ANCA but not MPO-ANCA (Figure 5B).

We next assessed the effect of plasma from patients with AAV on mPR3 using HC neutrophils. To exclude quantitative AAT effects, we selected the few plasmas from patients with active PR3-AAV and HCs with similar AAT levels (Figure 5C). mPR3 depletion was less efficient when using plasma from patients with active PR3-AAV, whereas active MPO-AAV, or remission AAV, and HC plasma all showed similar effects (Figure 5D). We reasoned that (a) either inflammatory mediators in plasma from patients with active PR3-AAV led to higher mPR3 levels, or that (b) AAT protein was modified, affecting its capacity to bind and consequently deplete PR3 from the neutrophil membrane, or that (c) both occurred. Spiking HC plasma with inflammatory mediators at picomolar concentrations, as found in patients with active PR3-AAV (36–38), increased mPR3, and this effect was further accelerated by PR3-ANCA but not MPO-ANCA (Figure 5E). These findings show that disease-characteristic inflammatory mediators counteract the mPR3–lowering AAT effect, thereby increasing mPR3 in the presence of identical AAT concentrations.

**Figure 6:**
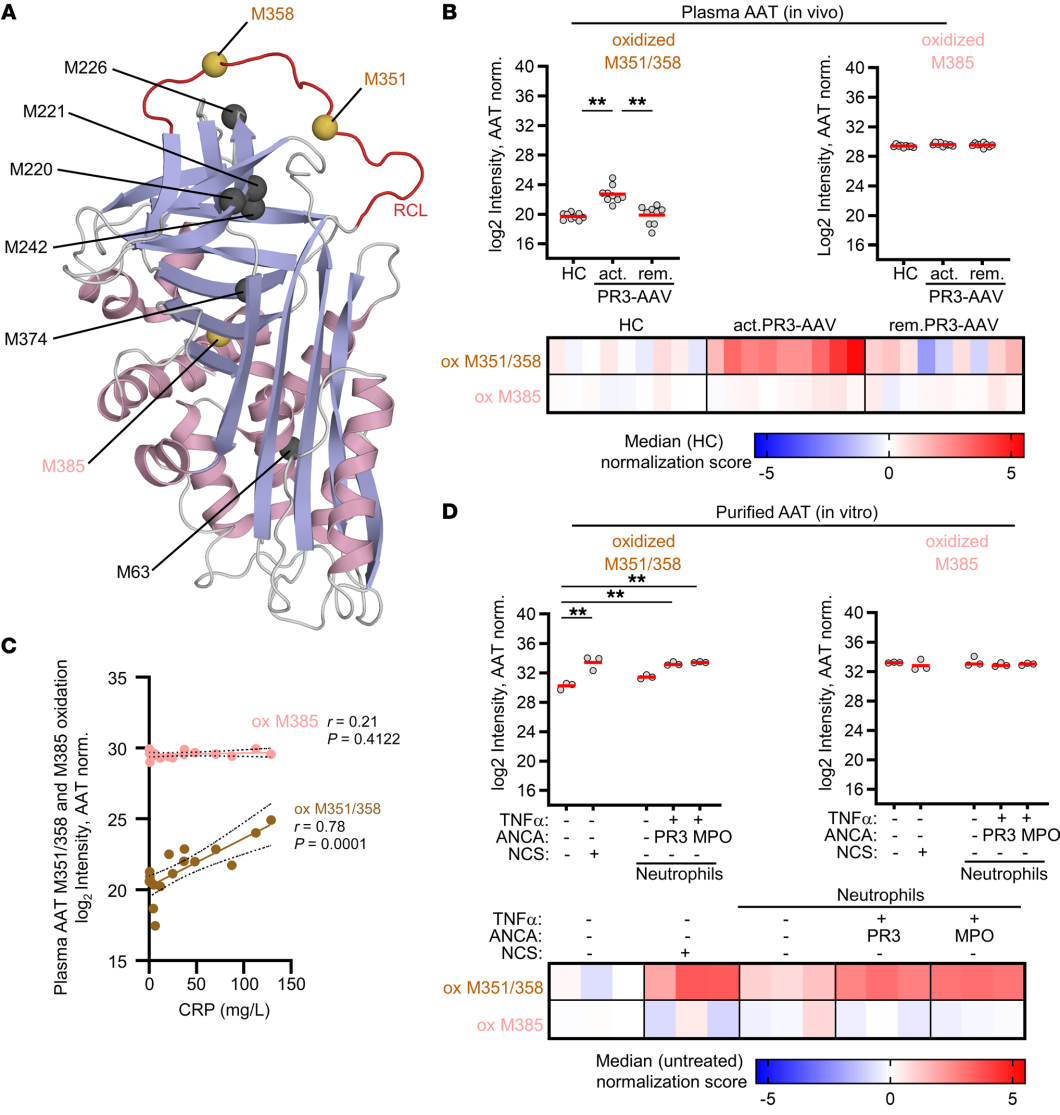
AAT methionine oxidation at positions M351 and M358 is increased in the plasma of patients with active PR3-AAV and in the presence of ANCA-activated neutrophils in vitro. (**A**) The AAT structure is depicted to illustrate the surface-exposed methionines (M) at positions 351 and 358 in the reactive center loop (shown in red), and the nonexposed M385. The structure is colored according to its secondary structure elements (helix, light magenta; β strands, light blue; loops, gray). Structural figure based on Protein Data Bank entry 1ATU and were prepared with PyMOL (Schrödinger) software. (**B**) Plasma from HC, patients with active PR3-AAV, and patients in PR3-AAV remission (*n* = 9/group) was analyzed for AAT site-specific methionine oxidation by PRM. M351/M358 double oxidation was significantly increased in plasma AAT from patients with active PR3-AAV, whereas M385 oxidization was not changed. Scatter plot (top) shows AAT protein normalized log2 intensities of oxidized peptides. For each group, individual patient data points are shown as circles, and the mean is indicated as a red line. The heatmap (bottom) displays the median normalization scores of the intensity data. (**C**) M351/M358 double oxidation of plasma AAT correlated with the inflammation marker CRP in plasma from patients with PR3-AAV (*n* = 18). (**D**) Purified WT-AAT was exposed to resting HC neutrophils, or TNFα-primed neutrophils stimulated with PR3- or MPO-ANCA IgG, respectively. After 30 minutes the reaction was stopped, and AAT was analyzed for methionine oxidation by PRM (*n* = 3/group). AAT exposure to ANCA-activated neutrophils resulted in strong increase in M351/M358 double oxidation, whereas M385 was not significantly affected. Intensities (log2) of oxidized peptides are normalized to AAT protein abundance and shown as individual data points for each treatment in the scatter plot with the red line indicating the mean value per group (top) and as heat maps of median normalization scores (bottom). (**B** and **D**)1-way ANOVA was performed with Tukey’s posthoc testing. *P < 0.05, **P < 0.01.

AAT oxidation, specifically oxidation of the 2 surface-exposed methionine (M) residues M351 and M358 in the reactive AAT center loop (Figure 6A), is a well-established mechanism that can reduce the inhibitory AAT activity toward NSPs at inflammatory sites (39, 40). Conceivably, oxidative stress in AAV could lead to AAT oxidation and could therefore impair mPR3 depletion by AAT. To determine whether this modification occurs in vivo, patient plasma was assessed by quantitative targeted mass spectrometry, namely Parallel Reaction Monitoring (PRM). We assessed unmodified and oxidized variants of peptides covering critical M351 and M358, and the unexposed M385 that is inaccessible to oxygen radicals as a control. We observed significantly increased M351 and M358 double oxidation in plasma from patients with active PR3-AAV that decreased with remission; this increase in oxidation was not seen for M385 (Figure 6B). These data indicate that this site-specific modification of AAT indeed occurs in vivo in patients with active PR3-AAV. Moreover, the level of M351/M358 double oxidation correlated with CRP, an indicator of systemic inflammation (Figure 6C). Conceivably, oxidative AAT modification is even more relevant in close proximity of ANCA-activated neutrophils. This notion is supported by our observation that M351/M358 double oxidation of WT-AAT strongly increased in the presence of ANCA-activated neutrophils and N-chlorosuccinimide (NCS) (41) that served as positive control (Figure 6D). Again, oxidation of M385 was not affected. The estimated ratio between oxidized and unmodified M351/M358 was much higher after in vitro exposure of AAT to activated neutrophils than in plasma samples from patients with active PR3-AAV (Supplemental Figure 2).

**Figure 7:**
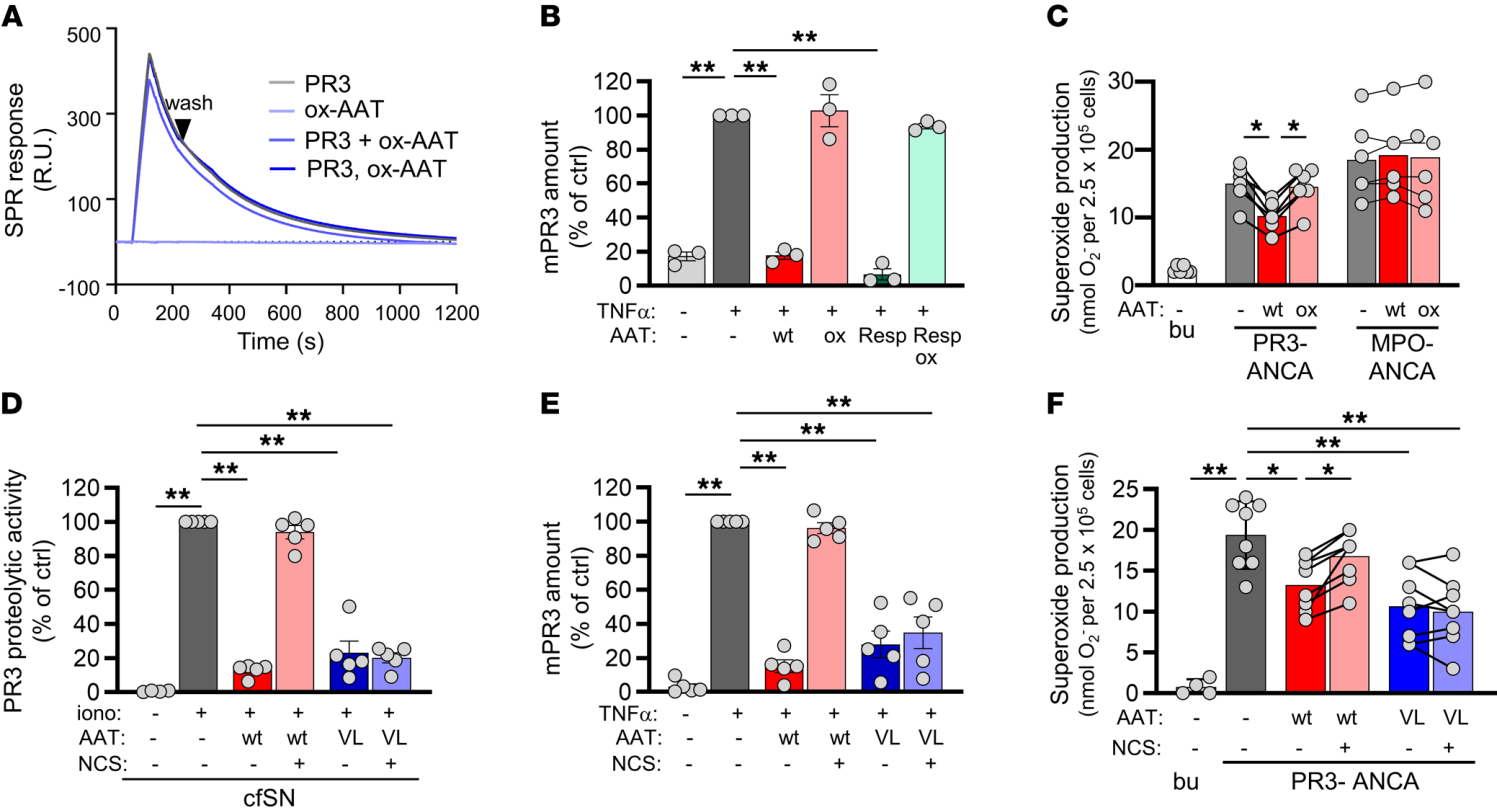
Methionine oxidation at position M351 and M358 abolishes AAT binding to PR3 and AAT-mediated protection from PR3-ANCA mediated neutrophil activation. (**A**) WT-AAT was exposed to the oxidant NCS. OX-AAT showed strongly reduced competition with CD177 for PR3 binding by SPR. At 0.25 μM, (**B**) oxidized WT- and EMA-approved respreeza(resp)-AAT caused no mPR3 reduction on neutrophils by flow cytometry (*n* = 3/group), and (**C**) Adding oxidized WT-AAT resulted in less PR3-ANCA–induced neutrophil activation by superoxide release (*n* = 3 independent experiments, using 4 different ANCA preparations). (**D**–**F**) At 0.25 μM oxidation-resistant VL-AAT (VL) exposed to NCS reduced (**D**) PR3 FRET activity (*n* = 5/group), (**E**) mPR3 on primed neutrophils (*n* = 5/group), and (**F**) PR3-ANCA induced superoxide release (*n* = 4 independent experiments, each using 2 different ANCA preparations). Individual results and the mean ± SEM are given. 1-way ANOVA (**B, D** and **E**), repeated measures 1-way ANOVA (**C** and **F**) within each group was performed with Tukey’s posthoc testing. *P < 0.05, **P < 0.01.

In contrast to unmodified WT-AAT, chemically oxidized WT-AAT (OX-AAT) did not form complexes with purified PR3, did not abrogate proteolytic PR3 activity (Supplemental Figure 3), and did not compete with CD177 for PR3 binding by SPR (Figure 7A). Importantly, OX-AAT, produced from recombinant WT-AAT or commercially available respreeza, a drug that is repetitively given to patients with AAT-deficiency, did not diminish mPR3 or neutrophil activation by PR3-ANCA (Figure 7, B and C).

Finally, we produced a recombinant AAT containing valine and leucine substitutions (VL-AAT) for the critical, oxidation-susceptible methionine residues in the reactive center loop (M351V/M358L) (42). This oxidation-resistant VL-AAT preserved its capacity to reduce proteolytic PR3 activity, mPR3 on neutrophils, and respiratory burst in response to PR3-ANCA, even in the presence of NCS oxidant (Figure 7, D–F). Respiratory burst inhibition was dose-dependent over a range of 0.1–10 μM (Supplemental Figure 4). These findings show that critical methionine-oxidation in the AAT center loop, found in patients with active PR3-AAV, compromises protective AAT effects, and that protection was preserved with oxidation-resistant VL-AAT, even when exposed to strong oxidative stress.

## Discussion

We explored alterations in the PR3 autoantigen and its major inhibitor, AAT, in AAV to define their roles in disease pathogenesis. We report several findings that we believe to be novel. First, PR3 and AAT were increased in patients with active AAV. This finding pertains to both PR3- and MPO-AAV. However, only in PR3-AAV did increased PR3 correlate with markers of systemic inflammation, kidney injury, and autoimmunity. Second, AAT diminished the binding of PR3 to the CD177 receptor on the neutrophil surface, thereby reducing neutrophil activation in the presence of PR3-ANCA but not MPO-ANCA. Third, disease modifiers, including inflammatory mediators and oxidative AAT modification, reduced neutrophil mPR3 depletion by AAT in active PR3-AAV. Substitution of critical methionine residues in the reactive AAT center loop preserved the protective AAT effects even when exposed to oxidants.

Associations between PR3-AAV and SNPs near or within the *PRTN3* gene were reported from different patient cohorts (25, 26). Some of these *PRTN3* SNPs provided an eQTL affecting neutrophil PR3 transcription (26), and reactivated PR3 transcription was reported in patients with active AAV (31, 43). In addition, some of the *PRTN3* SNPs from the AAV GWAS provided a pQTL that associated with increased plasma PR3 in HCs (27). However, whether patients with active AAV harbor increased neutrophil PR3 protein was not investigated in these studies. We provide what we believe to be novel information, namely that the PR3 protein content of a single neutrophil has approximately doubled in both active PR3- and MPO-AAV. The total PR3 blood pool, but also the individual neutrophil and plasma PR3 pools, correlated with clinical inflammation and kidney injury markers in PR3-AAV but not MPO-AAV. Increased PR3 protein may be linked to the PR3-AAV pathogenesis by various effects. For example, more mPR3 on the neutrophil surface leads to more PR3-ANCA binding, resulting in stronger neutrophil activation. Increased neutrophil PR3 increases the caspase 1–independent IL-1β generation, the cleavage of various extracellular substrates, and vascular damage (23, 24). Plasma PR3 correlated with the PR3-ANCA titer, conceivably by providing more autoantigen for the autoimmune response, particularly in individuals with a susceptible HLA-DP (25, 26) background.

Plasma AAT is the major PR3 inhibitor and is lowest in homozygous carriers of the z-allele. PR3-AAV associations with SNPs in the *SERPINA1* gene that were in linkage disequilibrium with the z allele were found in GWAS, suggesting a link between AAT levels and disease risk (25, 26). However, the mere presence of AAT deficiency did not increase the prevalence of PR3-ANCA and PR3-AAV as observed in patients with AAT deficiency–related chronic obstructive lung disease supporting the importance of the HLA context (44). Our data might seem paradoxical at first, as plasma AAT levels were found to be increased in the vast majority of patients with active PR3- and MPO-AAV, with only 1 patient with remission PR3-AAV with low AAT plasma levels. AAT is mainly produced by the liver, and levels increase with the acute-phase inflammatory response. Accordingly, we expected to observe increased AAT plasma concentrations in patients with active AAV, and positive correlations between the systemic inflammation marker CRP and AAT, as described by others (45). However, what was less expected was the increase of PR3 in plasma that exceeded the increase in plasma AAT on a molar ratio. Plasma AAT was still in excess compared with plasma PR3. Nevertheless, this subtle imbalance favoring PR3 has implications for the local neutrophil environment when cells release PR3 and other NSPs into their surroundings. This assumption is supported by a study on AAT and neutrophil elastase that measured and modeled AAT- and neutrophil elastase-dependent quantum proteolytic events and areas around neutrophils in human serum (46).

PR3-ANCA binding to PR3 on the neutrophil cell membrane and the subsequent neutrophil activation are central to the PR3-AAV disease concept. We confirm that the mPR3^hi^ neutrophil percentage and the amount of mPR3 are increased in PR3-AAV, and that the latter, but not the former, depends on disease activity (4, 8, 32). The high-affinity PR3 receptor CD177 followed the same membrane expression pattern as mPR3. Triggered by the observation that plasma AAT correlated inversely with mPR3 in HCs but not in our cohorts of patients with AAV, we performed mechanistic studies to test the hypothesis that AAT depletes mPR3 and that disease-modifiers counteract this effect. We found that increasing AAT concentrations progressively reduced the neutrophil mPR3 amount. The effect was reversible under inflammatory conditions when AAT was removed, mimicking emigration from the plasma to extravascular inflammatory sites. We provide what we believe to be novel mechanistic evidence that AAT binding to PR3 prevented PR3 binding to the CD177 neutrophil surface receptor. Moreover, CD177-bound PR3 dissociated from CD177 in the presence of AAT. Critically, this effect specifically protected against PR3-ANCA but not against MPO-ANCA–induced neutrophil activation, clearly separating the pathogenesis of the 2 conditions. However, we observed that the inverse correlation between mPR3 and AAT seen in HC was compromised in both patients with active and remission AAV. The fact that some patients with remission PR3- and MPO-AAV, despite a Birmingham Vasculitis Activity Score Version 3 (BVAS) of 0, still had slightly elevated CRP levels (Supplemental Table 1) and persistent low-titer ANCAs suggests that some residual inflammation may explain this observation (Supplemental Figure 6).

We observed that plasma from patients with active PR3-AAV caused less mPR3 depletion compared with plasma from HCs and patients with MPO-AAV, despite having similar AAT concentrations. Disease-associated cytokines, together with ANCA, contributed to this effect. In addition, we identified AAT oxidation as an AAV-related modifier that weakened the mPR3-lowering effect of AAT. Oxidative inactivation of the AAT antiproteinase activity in the respiratory tract of smokers was implicated in emphysema (47, 48). AAT harbors 9 total methionines, with both M351 and M358 located in the reactive center loop and responsible for NSP binding. M351 and M358 are exposed on the surface of the molecule and are therefore susceptible to methionine sulfoxide oxidation, resulting in inactivation of AAT (40). Activated neutrophils are an important ROS source and we reasoned that AAV, a neutrophil-mediated inflammatory condition, leads to oxidation-induced AAT inactivation. We found significantly increased M351 and M358 double oxidation in plasma AAT of patients with active PR3-AAV. The level of oxidation correlated with the inflammation marker CRP, suggesting that the observed modification was caused by systemic inflammation. This notion is further supported by the fact that ANCA-activated neutrophils caused strong M351 and M358 double oxidation in vitro.

**Figure 8:**
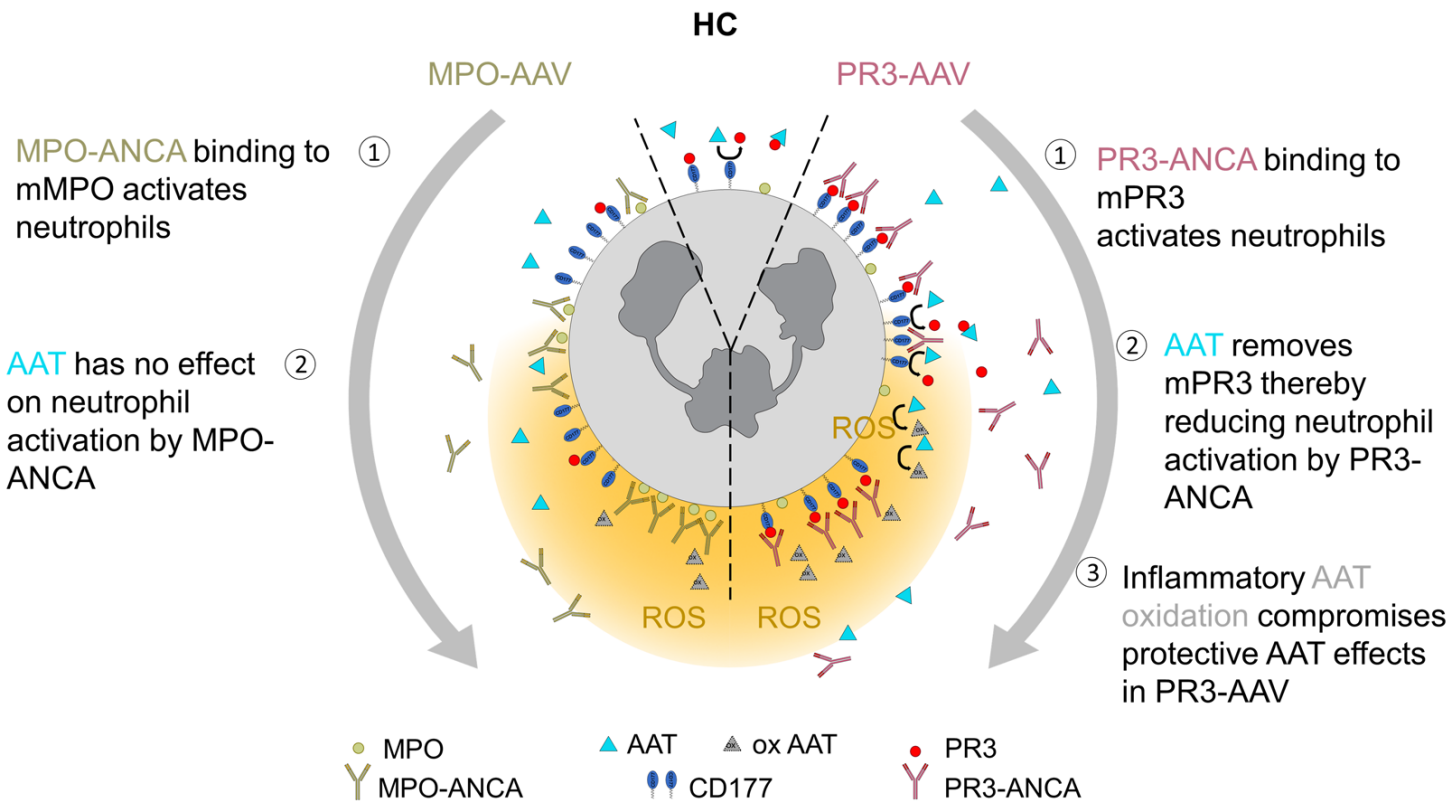
Antiproteinase-related AAT effects are protective in PR3- but not MPO-AAV. (1) Binding of PR3-ANCA (right side) and MPO-ANCA (left side) to their target antigens on the cell membrane activates neutrophils. (2) AAT decreases mPR3, thereby protecting from neutrophil activation by PR3- but not MPO-ANCA. (3) Oxidative AAT modification compromises the protective antiproteinase AAT effect in PR3-AAV.

We provide firm evidence that critical methionine oxidation is responsible for the reduced capacity of oxidized AAT to neutralize proteolytic PR3 activity, neutrophil mPR3, and diminished neutrophil activation by PR3-ANCA. M351V/M358L–VL-AAT preserved all these protective effects even when exposed to a strong oxidant. Our findings are consistent with recent studies showing that mice expressing high levels of the VL-AAT variant were protected against an excess of oxidants in vivo (42). A schematic illustrating key findings of protective AAT effects in the AAV context is shown in Figure 8. Our data support the notion that quantitative AAT aspects do not sufficiently reflect the complex AAV disease situation and that additional qualitative AAT aspects and the balance with PR3 are important. In addition to oxidative modification, AAT polymerization occurs in patients with AAT deficiency alleles leading to proinflammatory AAT properties that concern both PR3- and MPO-AAV (49). This state-of-affairs may explain why patients with AAV with a z- or s-alleles showed more intra-alveolar hemorrhage independent of the ANCA subtype (50).

In summary, our data indicate an increased PR3 pool in patients with active AAV that correlates with important clinical disease features. AAT exerts concentration-dependent protective antiproteinase effects in PR3-AAV that are compromised by oxidative modifications. An oxidation-resistant AAT preserved protection, despite oxidative stress, and could possibly be explored as an adjunct for current AAV-therapeutic strategies.

## Methods

### Patients and healthy individuals.

Patients with AAV, based on Chapel Hill Consensus Conference criteria (51), were recruited in the Department of Nephrology and Medical Intensive Care, Charité — Universitätsmedizin Berlin, Department of Nephrology, Helios Klinikum Berlin-Buch, and the Department of Nephrology, Endocrinology and Diabetology, Ernst von Bergmann Klinikum. Disease activity was assessed by the BVAS (52), and patients were grouped according to ANCA specificity (PR3-ANCA versus MPO-ANCA) and disease activity (active versus remission). Between January 2020 and July 2021, a total of 50 HC and 114 patients with AAV were recruited. All patients were ANCA positive and had generalized disease. At the time of blood sampling, 11 of 37 patients with active PR3-AAV were treatment naive, 25 of 37 had received steroids for less than 10 days, and 12 of 37 had received the first dose of i.v. cyclophosphamide or rituximab. The numbers for patients with active MPO-AAV were 10 of 22, 10 of 22, and 3 of 22, respectively. For patients in remission, 21 of 39 PR3-AAV were on steroids and 20 of 39 on azathioprine or rituximab maintenance. The numbers for patients in MPO-AAV remission were 11 of 16 and 5 of 16, respectively. Clinical and routine laboratory data were collected, and samples were assessed based on material availability. Of patients with AAV, 7 of 114 had total leukocyte but no neutrophil counts. For these patients, neutrophil numbers were calculated from the measured total leukocyte count using the average neutrophil percentage derived from the corresponding disease group. Population characteristics together with clinical and laboratory information are summarized in Supplemental Table 1.

### Preparation of neutrophils, neutrophil lysates, and plasma from human blood samples.

Venous heparinized blood samples were divided for neutrophil isolation and plasma preparation. Neutrophils were isolated using density-gradient centrifugation as previously described (6). For preparation of neutrophil lysates, 1 × 10^6^ neutrophils were resuspended in 15 μL lysis buffer supplemented with protease inhibitors (1% NP40, 0.1 mM quercetin, 10 μg/ml leupeptin, 10 μg/ml aprotinin, 5 mM iodoacetamid, 20 mM NaF, 1 mM PMSF, 0.2 mM Na_3_VO_4_ Sigma-Aldrich), followed by centrifugation at 18,000*g* for 10 minutes at room temperature. Cell-free supernatants were stored at –80°C until analyzed. Plasma was collected after blood centrifugation at 1,250*g* for 10 minutes at room temperature and stored at –80°C.

### Preparation of human IgG.

Normal- and ANCA-IgG were prepared from HCs and patients with active MPO- and PR3-ANCA disease using a High-Trap-protein-G column in an Akta-FPLC system (Cytiva Europe GmbH).

### PR3 and AAT mRNA expression in isolated neutrophils.

Total mRNA was extracted from 5 × 10^6^ neutrophils using 500 μL QIAzol and the RNeasy Purification kit (Qiagen). RNA was treated with deoxyribonuclease I (Qiagen) and cDNA was prepared using hexanucleotide primers and RevertAid First Strand cDNA Synthesis Kit following the manufacturers protocol (Thermo Fisher Scientific). For quantitative RT-PCR *Taq*Man technology (Thermo Fisher Scientific) was used with oligonucleotides and probes for human *PR3* (forward primer 5′-TGTCACCGTGGTCACCTTCTT-3′, reverse primer 5′-CCCCAGATCACGAAGGAGTCTAT-3′, 6-FAM 5′-TTGCACTTTCGTCCCTCGCCG-3′), human *AAT* (forward primer 5′- TGGATTTGGTCAAGGAGCTT-3′, reverse primer 5′-GTCCTCTTCCTCGGTGTCCT3-3′) and human *18S* (forward primer 5′-ACATCCAAGGAAGGCAGCAG-3′; reverse primer 5′-TTTTCGTCACTACCTCCCCG-3′; 6-FAM 5′-CGCGCAAATTACCCACTCCCGAC-3′); TaqMan Fast Universal PCR Master Mix and Fast SYBR Green Master Mix (Applied Biosystems, Thermo Fisher Scientific). Each sample was measured in duplicate and expression levels were normalized to *18S* expression. Quantification was performed using an Applied Biosystems 7500 Sequence detector and data were analyzed using SDS 7500 software and ΔΔCt comparative analysis as described by Applied Biosystems (Applied Biosystems, Thermo Fisher Scientific).

### Expression and purification of recombinant AAT species.

WT-(human M1 [V213]) and MUT-AAT (A355D/I356P/P357D/M358S) were produced from plasmid pTT5 (National Research Council [NRC], Canada) which carried the reading frames of an Igk chain–secretion signal and a C-terminal His tag. Introduction of the cDNA modification of MUT-AAT was described previously (33). In addition, an oxidation-resistant AAT variant was produced with amino acid substitutions replacing M351 with valine (V) and M358 with leucine (L). The VL-AAT plasmid was a gift from Ron Crystal (Cornell University, Ithaca, NY, USA) and was previously described (42). We amplified a C-terminal VL-AAT part containing the oxidation-resistant methionine substitutions in the reactive center loop (M351V/M358L) using the forward primer-*EcoRV* 5′-CTAGACGATATCATCACCAAGTTCCTGGAAA-3′ and the reverse primer-*AgeI* 5′-TTACTAACCGGTCTTTTGCGTCGGATTGACGA-3′ and subcloned this c381 bp product into the aforementioned WT-AAT (human M1 [V213]) using the restriction enzymes *EcoRI* and *AgeI*. This pTT5-AAT plasmid with the substitutions [V213, V351, L358] was confirmed by sequencing. The recombinant AAT proteins were expressed in human embryonic kidney (HEK) 293-6E cells (NRC, Canada) by transient transfection as described (53). Briefly, cells were cultured in suspension in Freestyle 293 Expression Medium (Invitrogen) supplemented with 0.1% Pluronic F-68 (Thermo Fisher Scientific), 25 μg/ml geneticin G-418 (Roche), and 0.5% Bacto TC Lactalbumin Hydrolysate (BD Biosciences) and purified from the culture supernatant by passage over a Ni-sepharose column (GE Healthcare).

### Chemical and neutrophil-induced AAT oxidation.

For chemical oxidation, 400 μL of 8 μM WT-, MUT-, or VL-AAT were incubated with 1.6 mM NCS oxidant (Aldrich) in 0.1 M Tris, pH 8.0 for 20 minutes at room temperature. After 10 minutes at room temperature, the reaction was quenched by adding 9.6 mM L-methionine. ZEBA-spin columns (Thermo Fisher Scientific) were used for buffer exchange to PBS. Protein integrity was checked by gel electrophoresis and Coomassie staining.

For neutrophil-induced AAT oxidation, 0.25 mM AAT was incubated in buffer containing 2 × 10^6^ neutrophils. Neutrophils were then left untreated or were primed with 2 ng/ml TNFα prior to adding 75 μg/ml PR3- or MPO-ANCA IgG, respectively. After 30 minutes, the reaction was stopped, and samples were analyzed using parallel reaction monitoring to detect methionine oxidation.

### Assessment of methionine oxidation using parallel reaction monitoring.

For sample preparation, AAT in vitro assay samples were resolved in lysis buffer (with a final concentration of 1% sodium deoxycholate, 10 mM dithiothreitol, 40 mM chloroacetamide, and 1 mM EDTA[all from Sigma-Aldrich], 150 mM NaCl, 50 mM Tris-HCl, pH 8) and heated for 10 minutes at 95°C. 100 ng Trypsin (Promega) and LysC (Wako) were added to each sample and digested for 5 hours at 37°C. For human plasma samples, a 1:10 dilution (in water) was prepared and 10 μL were used for digestion. 2× lysis buffer (2% sodium deoxycholate, 20 mM dithiothreitol, 80 mM chloroacetamide, 2 mM EDTA, 300 mM NaCl, 100 mM Tris-HCl, pH 8.0) was added to each sample, followed by incubation at 95°C for 10 minutes. The digest was performed with 1 μg Trypsin and LysC for 5 hours at 37°C. The digestion was stopped by acidifying each sample to pH <2.5 with 10% formic acid. After centrifugation to pellet insoluble material (21,000*g***)**, 10 minutes) the peptides were extracted and desalted using stage tip protocol (54).

For PRM, peptides for targeted analyses were selected based on methionine containing sequence and on abundance and scores (unmodified AAT control peptides) in human plasma shotgun proteome data. Charge state, retention time, and optimal collision energy for each peptide was defined with AAT in vitro samples, and the retention time window was set to 20 minutes for analytical runs. Peptide samples were separated on a reverse-phase column (20 cm fritless silica microcolumns with an inner diameter of 75 μm, packed with ReproSil-Pur C18-AQ 1.9 μm resin (Dr. Maisch GmbH) using a 98 minute gradient with a 250 nL/minute flow rate of increasing Buffer B concentration (from 2%–60%) on a HPLC system (Thermo Fisher Scientific) analyzed on a Q-Exactive HFx (Thermo Fisher Scientific). The PRM settings were, 30,000 resolution; 2 × 10^5^ AGC target; 1.6 m/z isolation window; and 60 ms maximum ion injection time. Data analyses were carried out using Skyline software (55). Peaks were manually selected based on retention time and dot product and the total intensity value of the correct peak area was extracted for peptide quantitation. The median intensity values of 7 unmodified AAT control peptides were used for normalization of the methionine containing peptide pairs (unmodified and methionine oxidized). Peptides used in PRM analyses are provided in Supplemental Table 2.

### SDS-PAGE and immunoblotting analysis.

Immunoblotting was performed as described before (23) using mAb to human PR3 (EPR6277, Abcam), mAb rabbit anti-AAT (EPR9090-71, Abcam), polyclonal rabbit anti-actin (13E5, Cell Signaling Europe) together with corresponding secondary antibodies, including polyclonal rabbit anti-mouse IgG-HRP (P026002-2, Agilent Technologies) and polyclonal donkey anti-rabbit IgG-HRP(NA934, Thermo Fisher Scientific) and visualized by enhanced chemiluminescence (Thermo Fisher Scientific).

### Assessment of PR3-, neutrophil elastase-, and cathepsin G-specific proteolytic activity by FRET.

For assessing specific proteolytic activity for PR3, NE, and CatG, 100 μL of cfSN plus 50 μL HBSS buffer were incubated with 20 μM selective FRET substrates as described (24). The selective FRET substrates for human NSPs were PR3: 2-Abz-VAD-(nor)V-ADYQ-EDA-Dnp; NE: 2-Abz-APEEIMRRQ-EDADnp; and CatG: 2-Abz-EPFWEDQ-EDA-Dnp. Fluorescence was monitored over 45 minutes using a plate reader (excitation 320 nm, emission 420 nm, SpectraMax i3x [Molecular Devices]). The proteolytic activity is reported as percent of control.

### Measurement of superoxide release.

Superoxide was measured using SOD-inhibitable ferricytochrome C reduction as described previously (32). Briefly, 7.5 × 10^6^ neutrophils were pretreated with 5 μg/mL cytochalasin B for 15 minutes on ice, primed with 2 ng/mL TNFα, and incubated with buffer, WT-AAT, MUT-AAT, or VL-AAT, as indicated. After 15 minutes, normal IgG, PR3-ANCA IgG, MPO-ANCA IgG (each 75 μg/mL), isotype control, mAb to PR3 (clone 43), or a mAb to MPO (Acris) (each 5 μg/ml) was added Experiments were performed in 96-well plates at 37°C for up to 60 minutes, and sample absorption with and without 300 U/mL SOD was measured at 550 nm in a microplate reader (Molecular Devices).

### Measuring mPR3 on the neutrophil membrane by flow cytometry.

1 × 10^6^ neutrophils were incubated in 100 μL HBSS with or without WT-AAT, MUT-AAT, VL- AAT, or in 100 μL diluted plasma from HC or patients with AAV, as indicated. When indicated, samples were stimulated with 2 ng/mL TNFα, a cytokine cocktail from 40 pg/mL TNFα, 30 pg/mL IL-6, 65 pg/mL GM-CSF, or 5 μg/mL mAbs to PR3 (clone 40) or MPO (Acris). Incubation was performed either in suspension conditions in tubes, on fibronectin-coated 24-well plates (Sarstedt), or on a confluent gMVEC (Cell Systems) monolayer for 30 minutes at 37°Cq. mPR3 was assessed by flow cytometry using the following mAbs to PR3, clone 43-8-3 and 81-3-3 (BioGenes), clone WGM-2-FITC (Abcam), clone MCPR3-2 (Thermo Fisher Scientific), with corresponding FITC–conjugated secondary anti-mouse IgG (Agilent). Double-staining for mPR3 and mCD177 was performed with Alexa Fluor 647–conjugated mAb anti-PR3 (clone 43-8-3) and Alexa Fluor 488–labeled mAb anti-CD177 (Biolegend). Additional surface proteins were stained with FITC–conjugated anti-CD11b, anti-CD18, or anti-CD66b mAbs (all from Beckmann Coulter). Cells were analyzed using a BD FACS Calibur or a BD FACS CANTO II (BD Biosciences) and FlowJo software (TreeStar).

### Biotinylation of cell-surface proteins.

5 × 10^6^ neutrophils were stimulated with 2 ng/mL TNF-α (30 minutes, 37°C) and incubated with cell impermeable EZ-Link Sulfo-NHS-SS-Biotin (Thermo Fisher Scientific) for 60 minutes on ice to biotinylate cell-surface proteins. After incubation with 0.25 μM WT- or MUT-AAT for 30 minutes, supernatants were collected and biotinylated proteins were pulled down with Dynabeads MyOne StrepAvidin T1 beads (Thermo Fisher Scientific), followed by electrophoresis and immunoblotting for PR3.

### Assessment of plasma and neutrophil PR3 by ELISA.

PR3 in human plasma and neutrophil lysates was assessed using a commercial PR3 ELISA (Elabscience Biotechnology Inc.), following manufactures instructions. Measurements detect both free and AAT-complexed PR3 (Supplemental Figure 5). The absorbance was determined at 450 nm in a plate reader (Molecular Devices). Neutrophil PR3 levels were normalized for μg neutrophil protein. The neutrophil PR3 pool was calculated from the PR3 amount per neutrophil multiplied by the number of circulating blood neutrophils in an estimated blood volume of 5 L. HCs were imputed to have normal values of 5 neutrophils/nL. The plasma PR3 pool was calculated from plasma volume × PR3 plasma levels. Plasma volume was calculated using the following equations: (23.7 × height [cm] + 9.0 × bodyweight [kg] – 1,709) × ([100 – {hematocrit [%] × 0.91}]/57.23) for men and (0.5 × height [cm] + 8.4 × weight [kg] - 4,811) × ([100 - {hematocrit [%] × 0.91}]/61.78) for women (56).

### Assessment of plasma AAT by nephelometry.

Plasma samples from HC and patients with AAV were analyzed for AAT protein by nephelometry on an Atellica Neph 630 platform (Siemens).

### Surface plasmon resonance.

Experiments were performed on a Biacore T200 instrument (GE Healthcare) using standard amine chemistry for coupling CD177 to the sensor chip (CM5, Cytiva Lifesciences). For kinetic measurements 5 μg/ml CD177 in sodium acetate, pH 4.5 was used with an immobilization level of approximately 770 counts. For wash experiments, 30 μg/ml CD177 in sodium acetate, pH 4.5 was injected for 420 seconds, resulting in an immobilization level of around 5800 counts. PR3 and AAT dilutions as soluble analytes were prepared in 10 mM HEPES, 150 mM sodium chloride and 0.05% Tween20 (Sigma-Aldrich) running buffer. The flow rate was 30 μL/minute and the assay temperature was 25°C.

### Statistics.

Results are given as means. Comparisons were made using ANOVA with Tukey’s posthoc analysis and Student’s *t* tests as indicated using GraphPad Prism8 software. P values of less than 0.05 were considered significant.

### Study approval.

The study was approved by the local ethic committee (Charité — Universitätsmedizin Berlin, Germany, EA4/025/18), and patients as well as HCs gave written, informed consent.

## Author contributions

ME and UJ contributed equally. ME collected and analyzed patient samples. UJ performed and analyzed mechanistic studies. RK and ADS conceived and designed the study. RK, UJ, CEG, ME, AS, DJ, ADS, MK, LK, KUE, and PM planned and guided experiments. ME collected human samples. ME, UJ, CEG, and MK analyzed human samples and performed experiments. RK, AS, LK, MB, and SE cared for the patients. RK wrote the manuscript, and all authors revised and approved the final version of the manuscript.

## Supplementary Material

Supplemental data

ICMJE disclosure forms

## Figures and Tables

**Table 1:**
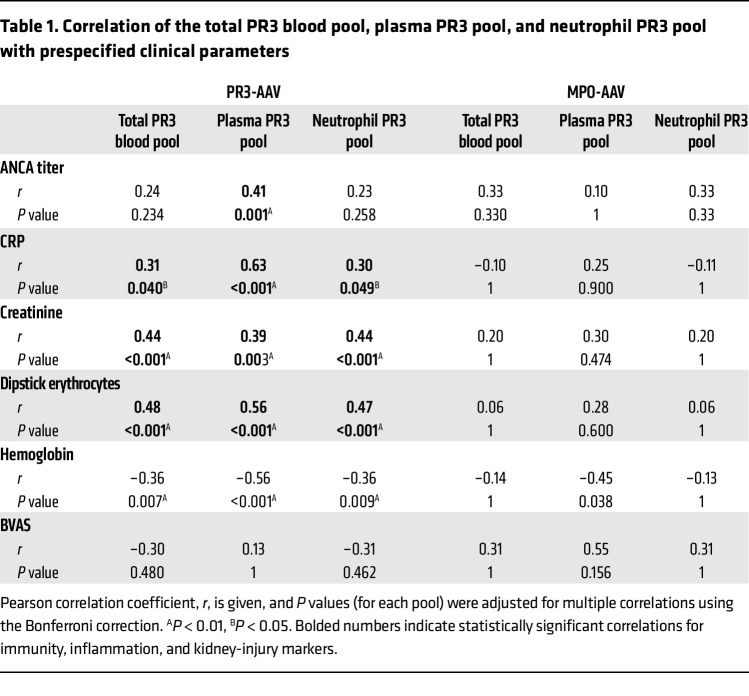
Correlation of the total PR3 blood pool, plasma PR3 pool, and neutrophil PR3 pool with prespecified clinical parameters

## Version 1 09/20/2022

In-Press Preview

## Version 2 12/01/2022

Electronic publication
