# Supplementary material for: Protective α1-antitrypsin effects in autoimmune vasculitis are compromised by methionine oxidation
Source: J Clin Invest. 2022 Dec 1;132(23):e160089. doi: 10.1172/JCI160089 (PMC9711875; doi:10.1172/JCI160089)
Supplement: Supplemental data [file jci-132-160089-s098.pdf]

Supplemental Figure S1

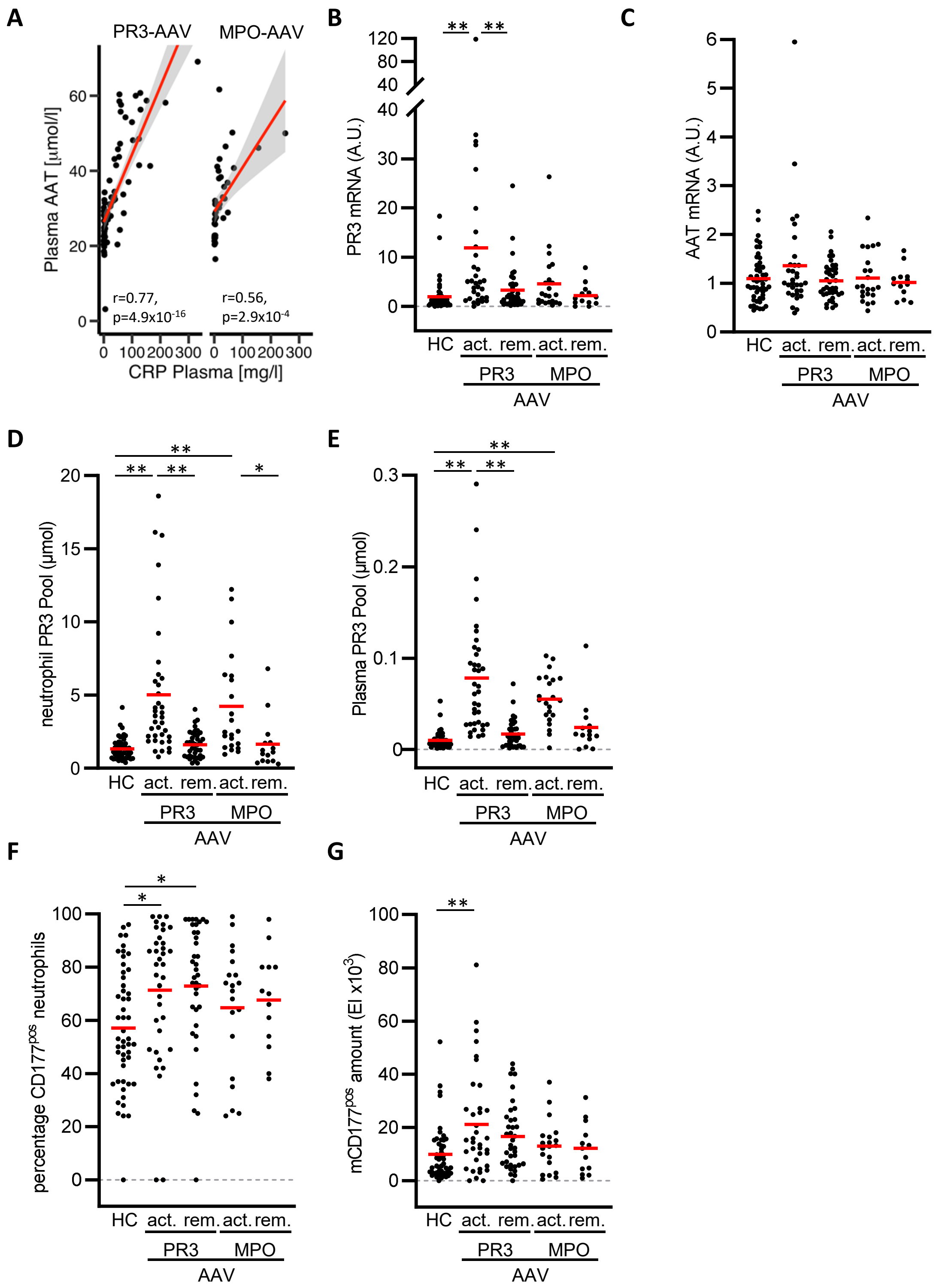

Supplemental Figure S1 (A) Plasma AAT correlation with plasma CRP in PR3- and MPO-AAV is illustrated. **(B)** PR3 mRNA and **(C)** AAT mRNA expression (n=50, 32, 39, 22, 13 from left to right). was measured by RT-PCR. **(D)** The neutrophil (n=50, 37, 39, 22, 15 from left to right) and **(E)** plasma PR3 pool was calculated in HC and AAV patients (n=50, 37, 39, 22, 16 from left to right). **(F)** The percentage of mCD177^{pos} neutrophils is depicted (n=50, 36, 37, 20, 13 from left to right). **(G)** The mCD177 amount is given as expression index of the mean MFI (MFI EI, n=50, 36, 37, 20, 13 from left to right). Individual results are depicted, and the mean is indicated. One-way ANOVA was performed with Tukey post-hoc testing. * is p<0.05, ** is p<0.01.

Supplemental Figure S2

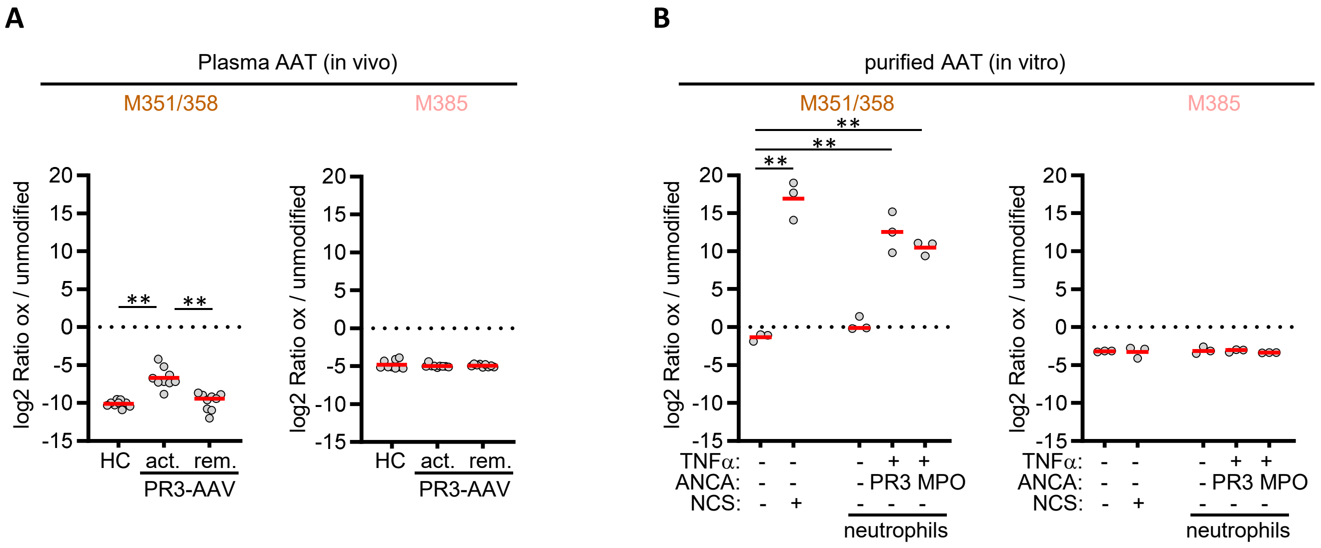

Supplemental Figure S2. Detection of oxidized M351, M358, and 385 by Parallel Reaction Monitoring (PRM). Log2 Ratio between oxidized and unmodified peptide pairs were calculated for **(A)** Plasma AAT (n=9/group), and **(B)** purified AAT (n=3/group). One-way ANOVA was performed with Tukey post-hoc testing. * is p<0.05, ** is p<0.01.

Supplemental Figure S3

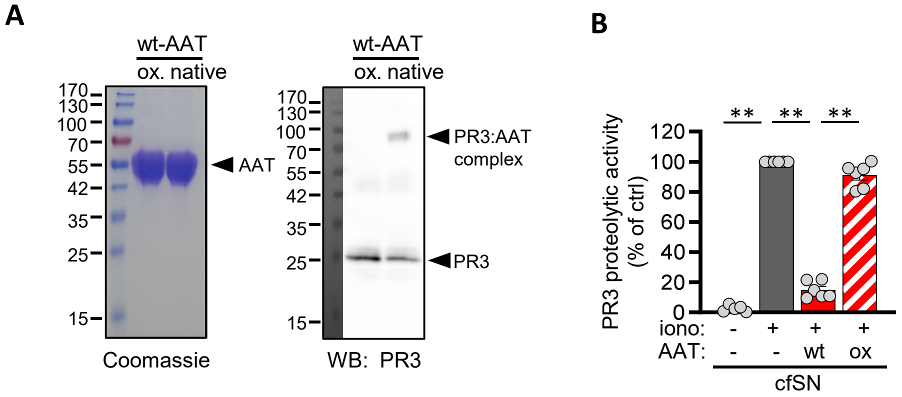

Supplemental Figure S3. Oxidized AAT does not bind and neutralizes proteolytically active PR3. wt-AAT was exposed to the oxidant N-chlorosuccinimide (NCS). **(A)** NCS treatment did not result in wt-AAT degradation as shown by electrophoresis and Coomassie staining but reduced complex formation with PR3 shown by anti-PR3 immunoblotting. **(B)** NCS-treated wt-AAT showed strongly reduced PR3 activity inhibition by FRET assay (n=6/group). Individual results and the mean ± s.e.m. are given. One-way ANOVA was performed with Tukey post-hoc testing. * is p<0.05, ** is p<0.01.

Supplemental Figure S4

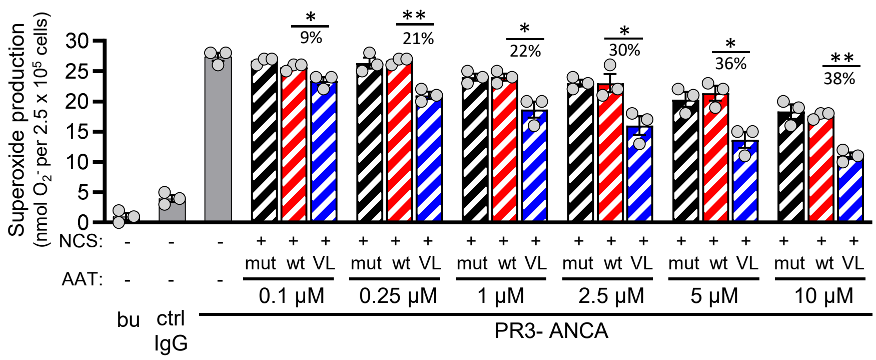

Supplemental Figure S4, VL- but not wt-AAT inhibits PR3-ANCA-induced respiratory burst in neutrophils even when exposed to a strong oxidant. Increasing concentrations of NCS-treated VL-AAT concentrations progressively inhibited superoxide release as indicated by the % inhibition (n=3/group), whereas NCS-treated wt-AAT did not differ from mut-AAT). Individual results and the mean ± s.e.m. are given. One-way ANOVA within the groups was performed with Tukey post-hoc testing. * is $p<0.05$, ** is $p<0.01$.

Supplemental Figure S5

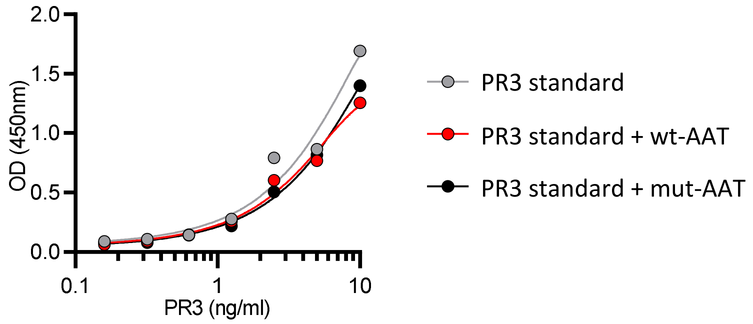

Supplemental Figure S5. The PR3 ELISA (Elabscience Biotechnology Inc.) detects free and AAT-bound proteinase 3. Addition of 0.25 μM wt- or mut-AAT to the manufacter's PR3 standard working solution that lacked AAT did not affect PR3 detection.

Supplemental Figure S6

A

Spearman correlation between
mPR3 amount and plasma AAT

HC (r= -0.32, p= 0.02)
active PR3-AAV (r= 0.21, p= 0.22)
remission PR3-AAV (r= 0.35, p= 0.04)
active MPO-AAV (r= 0.18, p= 0.45)
remission MPO-AAV (r= 0.08, p= 0.80)

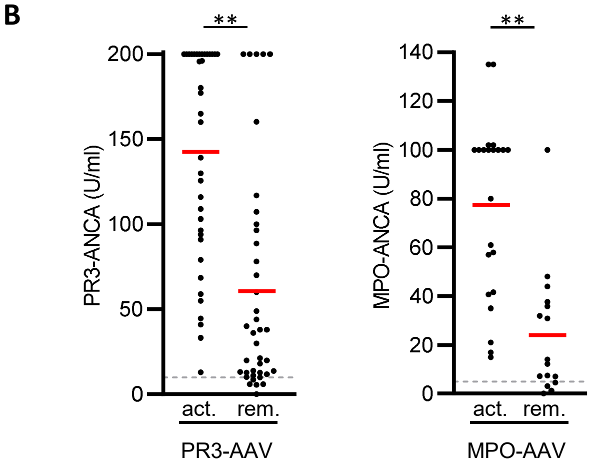

Supplemental Figure S6. Spearman correlation between mPR3 amount and plasma AAT and PR3- and MPO-ANCA titer in active and remission AAV patients. (A) The inverse correlation between mPR3 and AAT in HC was compromised in active and remission AAV patients. **(B)** PR3- and MPO-ANCA titer were measured for PR3- (active n=37, remission n=39) and MPO-ANCA patients (active n=22, remission n=16), respectively. Dotted lines represent the upper limit of the normal range. Individual results and the mean are given. Two-tailed Student's t-Test was performed. * is p<0.05, ** is p<0.01.

Supplemental Table 1

Demographic and clinical information of HC and AAV patients

	**Diagnosis**				
	**HC**	**active PR3-AAV**	**rem. PR3-AAV**	**active MPO-AAV**	**rem. MPO-AAV**
n	50	37	39	22	16
Age — year	53	63	64	66	60
Female —no. (%)	27 (54)	16 (43)	18 (46)	8 (36)	9 (56)
Male —no. (%)	23 (46)	21 (57)	21 (54)	14 (64)	7 (50)
**Disease entity**					
GPA	-	37	39	0	0
MPA	-	0	0	19	16
EGPA	-	0	0	3	0
**Clinical parameters**					
BVAS (0-63)	-	17±7	0	19±5	0
CRP [mg/l]	-	**76.4±64.8***	5.9±9.7	**39.5±58.2***	8.0 ± 14.24
Creatinine [mg/dl]	-	3.0±2.3	1.4±0.7	2.9±2.2	1.8±1.1
Hemoglobin [g/dl]	-	9.9±2.1	13.6±1.3	10.4±1.5	12.9± 1.9
Hematokrit [%]	-	**30±6▴**	41±4	**31±4▴**	38±5
Leukocytes [/nl]	-	13.6±6.0	8.4±2.3	10.7±4.3	8.4±2.8
Neutrophils [/nl]	-	**11.4±5.7***	5.9±2.1	**7.6±3.5***	5.6±2.3
Platelets [/nl]	-	427±214	271± 86	347±108	277± 82
**Organ involvement - no. (%)**					
Kidney	-	28 (76)	-	18 (82)	-
Lung	-	25 (68)	-	10 (45)	-
Ear/Nose/Throat	-	20 (54)	-	4 (18)	-
Muscle/Joints	-	12 (32)	-	3 (14)	-
Skin/Mouth/Eyes	-	11 (30)	-	0 (0)	-
Central nervous system	-	6 (16)	-	5 (23)	-

Data are presented as no or mean ± SD.

* indicates a significant difference between active PR3-AAV and active MPO-AAV as measured by two-tailed t-test.

▴ indicates a significant difference between active AAV and their corresponding remission group as measured by two-tailed t-test.

NA = not applicable

Supplemental Table 2

Peptides used in PRM analysis

Peptide sequence	m/z	Charge State	Analytical Group
GTEAAGAMFLEAIPMSIPPEVK	753.71821	3	unmodified M351/358
GTEAAGA(ox)MFLEAIP(ox)MSIPPEVK	764.38148	3	oxidized M351/358
SPLFMGK	390.20965	2	unmodified M385
SPLF(ox)MGK	398.207107	2	oxidized M385
AVLTIDEK	444.755475	2	unmodified control
VFSNGADLSGVTEEAPLK	917.465081	2	unmodified control
ITPNLAEFAFSLYR	821.435398	2	unmodified control
LSITGTYDLK	555.805697	2	unmodified control
QINDYVEK	504.753464	2	unmodified control
SASLHLPK	426.750527	2	unmodified control
SVLGQLGITK	508.310949	2	unmodified control
